# Tracking sustainability in crop pest management in the United States using an eco-efficiency index

**DOI:** 10.3389/finsc.2025.1582496

**Published:** 2025-05-20

**Authors:** Madison Love, Roger D. Magarey, Brian L. Holderman, Danesha Seth Carley, Federico Maggi, Naomi Singer

**Affiliations:** ^1^ Department of Horticultural Science, North Carolina State University, Raleigh, NC, United States; ^2^ Center for Integrated Pest Management, North Carolina State University, Raleigh, NC, United States; ^3^ School of Civil Engineering, The University of Sydney, Sydney, NSW, Australia; ^4^ Department of Statistics, North Carolina State University, Raleigh, NC, United States

**Keywords:** eco-efficiency, integrated pest management, pesticide risk assessment, pesticide use trends, sustainability, agricultural productivity, pesticide externalities, toxicity levels

## Abstract

While agricultural pesticides are considered essential for global food security, their use poses significant environmental and human health risks. Integrated Pest Management (IPM) offers a science-based framework to minimize these risks by integrating multiple pest management strategies. However, IPM adoption and funding in the United States have been limited, partly due to a 2001 government report highlighting the lack of measurable reductions in pesticide use. To address this challenge, we propose an index based on eco-efficiency, the ratio of agricultural productivity to environmental impacts, to quantify, track, and incentivize IPM adoption. Using crop production and pesticide use data, including both the mass and toxicity of active ingredients, eco-efficiency scores were calculated for ten major U.S. crop groups from 1992 to 2018. The results demonstrate the potential of this index to monitor progress over time and identify shifts in pesticide use relative to crop production. This approach offers a practical, data-driven tool to evaluate pesticide risk reduction, prioritize IPM research and Extension efforts, and support future policy and funding decisions aimed at promoting more sustainable agricultural practices.

## 1 Introduction

While the use of agricultural pesticides is important to global food security, their associated risks continue to pose significant challenges, particularly in terms of environmental impact and human intake via food residues (1–3). Despite an estimated biodegradation of 82% of applied pesticides, approximately 10% persists as environmental residue and around 7% leaches into aquifers (4), contributing to substantial pollution risks (5) and annual costs to human health and the environment estimated between 13 and 35 billion dollars (6, 7). While human health risks associated with organophosphates have likely decreased due to their declining use and replacement with alternative pesticides, concerns about human health and environmental impacts persist, as some organophosphates remain in use and newer alternatives, such as neonicotinoids and pyrethroids, present their own risks (8, 9). One additional critical area of concern is the effect of insecticides on pollinators and other beneficial arthropods which are essential for maintaining both natural and agricultural systems (10–12). Declining beneficial arthropod populations in part due to pesticide exposure may contribute to reduced crop yields, potentially impacting food security (13–18). In response to these concerns, there is increasing emphasis on evaluating and mitigating the risks pesticides pose to beneficial arthropods (19–23).

Due to public concern of the environmental impacts associated with pesticides, the Clinton Administration, with joint testimony made by the United States Department of Agriculture (USDA), Environmental Protection Agency (EPA), and the Food and Drug Administration (FDA), established a national goal in 1993 to implement Integrated Pest Management (IPM) practices on 75% of U.S. crop acreage by the year 2000 (24). Integrated pest management is a sustainable, science-based decision-making process that combines biological, cultural, physical and chemical tools to identify, manage and reduce risk from pests and pest management tools in a way that minimizes economic, human health, and environmental risks (25). Moreover, this multifaceted approach extends beyond pest management to encompass other factors in production including management, business, and sustainability while highlighting the essential role research and outreach contributes to advancing effective pest management practices (26). Unfortunately, the U.S. Government Accountability Office (GAO) reported in 2001 that IPM failed to demonstrate significant reductions in pesticide use and lacked methods for measuring economic and environmental impacts (24). Consequently, research and Extension efforts in IPM have faced dramatic funding cuts, dropping from $204 million in 1997 (27) to just $21 million allocated to the USDA National Institute of Food and Agriculture (NIFA) Crop Protection and Pest Management Program which is the only designated federal source of IPM funding for public research and extension. While recent studies have demonstrated increased adoption of specific IPM practices (28), these studies fail to quantify reductions in environmental and human health risks.

The agricultural community continues to face significant challenges in adopting IPM including the perception of high implementation costs, limited awareness, practical difficulties in execution, and a lack of sufficient incentives to drive widespread adoption (29, 30). Compared to conventional pest management programs that heavily rely on pesticides, IPM incorporates a broader range of strategies, requiring additional management practices that often demand greater knowledge, planning, and resources. Although biodiversity initiatives from major retailers, such as Walmart Inc., Whole Foods Market, Inc., and The Kroger Co., provide some motivation for IPM adoption, their impact is limited to growers within their supply chains. Additionally, these certifications primarily evaluate risk reduction qualitatively, focusing on changes in practices rather than employing quantitative metrics to measure changes to pesticide externalities. Furthermore, while IPM strategies can provide benefits for those who implement it (2, 31, 32), growers receive no direct compensation for the broader societal benefits, such as reduced pesticide externalities, generated by their efforts (29). Pesticide externalities encompass the unintended social, environmental, and economic consequences of pesticide use, such as water contamination, biodiversity loss, and human health risks, that are not accounted for in market prices (33, 34). Addressing these barriers is essential to advancing IPM adoption and realizing its potential to mitigate environmental and human health risks.

Some countries have sought to promote or mandate IPM adoption by setting pesticide reduction targets. Notably, the European Union (EU) introduced pesticide risk reduction initiatives under its Farm to Fork policy (35, 36), establishing legally binding national targets aimed at reducing pesticide use while enforcing stricter regulations to encourage environmentally responsible pest management. These measures were intended to ensure that farmers and professional pesticide users incorporated IPM strategies into their practices. In 2023, the EU went further by implementing a ban on all pesticide use in sensitive areas, including parks, greenways, and schools. However, despite these ambitious efforts, the proposed regulations sparked widespread protests from farmers, ultimately leading to their abandonment (37). This resistance underscores the challenges of imposing restrictive measures and highlights the need for alternative approaches. A program centered on incentives is more likely to drive investment in research and development for high-quality innovations compared to regulatory mandates (38). In contrast, mandates in the U.S. could face significant resistance from growers, similar to the pushback observed in the E.U. (37).

The current status of pest management in U.S. crop production has been described as the pesticide quandary (39). The term encapsulates a recurring cycle characterized by a lack of adoption of IPM, over-reliance on pesticides, public concerns about the environmental and health risks of pesticides, litigation, circumvention of regulatory systems, and the subsequent loss of critical pesticides essential for pest control. One possible method to escape the pesticide quandary is to follow a similar path that has been developed to address agriculture’s carbon footprint in mitigating climate change. Programs like the USDA Climate-Smart Agriculture and Forestry (CSAF) program aim to identify, quantify, track and incentivize carbon reduction strategies (40, 41). A similar strategic model could also be applied to pesticide use reduction, encouraging growers to adopt IPM practices.

This paper proposes the idea of a Pest-Smart Agriculture analog to identify, quantify, track and incentivize pesticide risk reductions. This approach would not replace IPM but serve as a complementary tool to enhance its adoption. By providing measurable metrics, Pest-Smart Agriculture could address the longstanding concerns outlined in the 2001 GAO report (24) regarding the absence of standardized metrics for evaluating IPM outcomes in terms of economic and environmental impact. Additionally, these metrics could serve as the foundation for risk-reduction programs supported by government funding or market-driven incentives, fostering a more sustainable and effective approach to pest management in agricultural systems.

The first step towards implementing Pest-Smart Agriculture is to quantify the risks associated with pesticide use. A promising approach for measuring and tracking pesticide risk reduction is the concept of Regulatory Threshold Levels (RTLs) and Total Applied Toxicity (9, 42). The RTL is a derived threshold that indicates potential biodiversity impacts based on data obtained from officially maintained regulatory databases from the EPA ECOTOX Knowledgebase (9). While internationally recognized RTLs for pesticides in drinking water have been established in countries such as the EU, U.S., Japan, and Canada (43), the U.S. EPA has not yet set RTLs specifically for agricultural pesticide use to our knowledge. Since the RTLs discussed in this paper lack an official regulatory status in the U.S., an equivalent term, “Toxicity Level”, will be used instead. Establishing standardized Toxicity Levels for agricultural pesticide use in the U.S. could provide a crucial foundation for advancing Pest-Smart Agriculture, enabling more precise risk assessments.

To evaluate Toxicity Levels, the Total Applied Toxicity, a Risk Quotient (RQ) that represents the ratio of an exposure level to a toxicity endpoint value (44), is used as a key toxicity measurement. In its simplest form, exposure is represented by the total amount of pesticide applied. However, as will be discussed later in this paper, this is a simplified approach that does not fully account for the complexities involved in pesticide risk assessment and can be further improved. Risk quotients are preferred over qualitative and semi-quantitative pesticide ranking methods, such as the Environmental Impact Quotient (EIQ), which are subject to both conceptual and mathematical limitations (45, 46). Risk quotient values provide a more robust framework by assigning small values to active ingredients with significant biodiversity impacts and larger values to those with lesser impacts. The Total Applied Toxicity serves as an indicator of potential pesticide impacts and is calculated by dividing the total applied mass of each active ingredient by its corresponding Toxicity Level. Further analysis is completed by calculating an eco-efficiency index for each crop based on crop production and annual pesticide use reports.

Eco-efficiency, defined as the ratio of productivity to the environmental impact of pest management (39), provides a valuable framework for assessing the sustainability of agricultural practices. This index offers several key advantages in quantifying and tracking pest management pesticide use as it relates to productivity and environmental impact. First, it accounts for fluctuations in pesticide use relative to crop production, offering a more precise and dynamic assessment of pesticide impacts over time. Second, by integrating both productivity benefits and environmental risks into a single metric, the eco-efficiency index helps balance the dual objectives of minimizing ecological harm while maintaining agricultural productivity. Completely eliminating pesticide use is not a viable solution, as it would leave essential food crops vulnerable to pests, posing serious threats to food security (3). ​​Instead, the eco-efficiency index provides a practical pathway toward sustainable pest management by promoting a balanced, data-driven approach that optimizes productivity while reducing environmental impact.

This study is a proof of concept that aims to demonstrate the use of eco-efficiency in evaluating the sustainability of crop pest management in the U.S. To achieve this, eco-efficiency indices were calculated for ten crop groups and seven species groups across the U.S. from 1992 to 2018. Each crop-specific index was designed to track changes in eco-efficiency over time, providing a dynamic assessment of progress within individual crops rather than a direct comparison between them. By examining eco-efficiency trends across multiple crops, this study offers valuable insights into the evolving relationship between pest management practices, productivity, and environmental impact. These findings establish a foundation for future discussions on research, extension, and policy initiatives aimed at improving eco-efficiency in agricultural pest management.

## 2 Materials and methods

Eco-efficiency indices were calculated for 10 crop groupings using a comprehensive dataset from multiple sources of data spanning from 1992 to 2018 (47). The applied mass of pesticides from 1992 to 2018 were sourced from the United States Geological Survey Pesticide National Synthesis Project (USGS/PNSP) within the North American Water Assessment of 2022. The data, which includes “high” and “low” annual application masses for each U.S. state from 1992 to 2018, were based on surveys conducted by the USDA National Agricultural Statistics Service (USDA/NASS) between 2007 and 2012 (47). For data outside of this period, interpolation and extrapolation methods outlined by Baker and Stone (48) were applied. The USGS/PNSP data covered six dominant crops including corn, soybean, wheat, cotton, rice, and alfalfa, as well as four aggregated crop classes: i) vegetable and fruits, ii) orchard and grapes, iii) pasture and hay, and iv) other crops, as detailed in **Supplementary Table S1** which expands upon the classes found in Maggi et al. (47). The total mass of pesticides applied was calculated by pesticide type for each crop category.

Total crop production for each of the 10 groups from 1992 to 2018 was estimated using publicly available data maintained by USDA National Agricultural Statistics Service (NASS) (**Supplementary Table S1**). Annual crop production estimates for corn, wheat, rice, pasture and hay, and alfalfa were obtained from the NASS “Quick Stats” database, which enabled targeted searches by commodity, category, measurement unit, and location. Data for orchard and grapes were sourced from the annual “USDA Citrus Fruits Summary” and “USDA Non-Citrus Fruits and Nuts Summary” reports, while vegetable and fruits primarily relied on the “USDA Non-Citrus Fruits and Nuts Summary” and “USDA Vegetable Summary” reports. Additional crops within this category not included in these reports, such as dry beans, peas, potatoes, and sweet potatoes, were gathered from the “Quick Stats” database. Production for each crop was reported in a variety of measurement units: corn, wheat, and soybeans were reported in bushels; cotton in 480-pound bales; rice in hundredweight (CWT); and aggregated pasture and hay and alfalfa in tons. Crop groups containing multiple crops, such as orchard and grapes, vegetable and fruits, and aggregated other crops, used a variety of measurement units. To maintain uniformity, all crop categories were converted to metric tons.

One challenge encountered in the analysis was the inconsistency in data reporting over time. From 1992 to 2015, vegetable production was recorded under the category “Principal Vegetable,” while from 2016 to 2018, it was reported as “Principal Vegetable Fresh Market and Processing,” leading to nearly double the reported total for these years. The most significant discrepancies in data reporting and categorization, including missing data for certain years, were observed in the vegetable and fruits and orchard and grapes categories. These issues are detailed in the **Supplementary Methods** and in **Supplementary Table S1**, specifically, the “Summary” worksheet tab.

Next, the TAT was calculated (Equation 1) using the mass of pesticide applied and the Toxicity Level values for each pesticide (**Supplementary Tables S3**, **S4**). The Toxicity Level represents a threshold indicative of potential biodiversity impacts, derived from EPA’s ECOTOX Knowledgebase (9). Toxicity Level values for 381 active ingredients were obtained (9) for various species groups, including terrestrial organisms such as mammals, plants, arthropods, and birds, as well as aquatic organisms including fish, plants, and invertebrates (**Supplementary Table S2**). Due to limited data and differences in toxicological measurement, pollinators were excluded from the analysis; however, the impact of pesticides on pollinators can be inferred from the Toxicity Level values for arthropods.

The Total Applied Toxicity (TAT) for each species group (s), year (y), and crop (c) was calculated by summing each of the 381 active ingredients’ applied mass in kg (m) divided by the Toxicity Level for each pesticide active ingredient (a) and species grouping(s) (Equation 1):


(1) $$\mathrm{TA}{T_{\mathrm{s,y,c}}}_{=\sum_{a}}\frac{M_{\mathrm{a,c,y}}}{TL_{\mathrm{a,s}}}$$

where *M_a,c,y_* is the mass (kg) of pesticide active ingredient (a), to crop group (c), applied in each year (y) and *TL_a,s_* is the toxicity level expressed in Toxicity units (Tu) such as mg/kg, mg/ha or mg/L depending on toxicity endpoint for active ingredient (a) and species grouping (s). The resulting units for TAT are kg of pesticide active ingredient/Tu. This formulation of the units preserves the linkages to the original toxicological studies.

Because of missing information, metam was assigned the same Toxicity Level as metam potassium, and Dichloropropene, a fumigant which represents a 5% of pesticide use, was mistakenly assigned the same Toxicity Levels as the herbicide 2,4-Dichlorophenoxyacetic acid (2,4-D). This error likely led to an underestimation of TAT in birds and mammals, particularly in non-row crops and cotton, which exhibited the highest usage. Additionally, the following pesticide active ingredients, accounting for 9% of all pesticide mass, were not included in the analysis: petroleum oil, petroleum distillate, and sulfuric acid. These active ingredients were considered to be either too variable or were not classified as conventional pesticides with available Toxicity Level values. After these adjustments, only 1.6% of the total applied mass was from active ingredients not available in the Toxicity Level database (9). A list of these pesticides as well as the usage of Dichloropropene by crop is included in the worksheet tab titled “Excluded from TAT” found in **Supplementary Table S3**. Another limitation of the pesticide use data set was that seed treatments were not included after 2014 (49).

Pesticide classes, including herbicides (amino acid synthesis inhibitors, cell membrane disruptors, growth regulators, lipid synthesis inhibitors, pigment inhibitors, photosynthesis inhibitors) (9), insecticides (carbamate, neonicotinoid, organochlorine, organophosphorus, pyrethroid) (9), and fungicides (as classified by the Fungicide Resistant Action Committee [FRAC]) (50), were used to estimate missing Toxicity Level values for specific species groups. If at least three pesticides within a class had available Toxicity Level values for a particular species group, the average of these values was applied to estimate the missing values for that class. If a Toxicity Level value was unavailable for a specific class and species group, all pesticide active ingredients within that class were excluded from the analysis. For each crop and species group, the proportion of applied mass that contained a Toxicity Level value was determined. For each species group, the proportion of active ingredients with an assigned Toxicity Level value was assessed. For terrestrial organisms, the percentages were mammals and plants 71.1%, arthropods 66.7%, and birds 74.6%. For aquatic organisms, the percentages were fish 89.3%, plants 79.6%, and invertebrates 96.9%. For each crop group, the percentage of applied mass for which the pesticides active ingredients had a Toxicity Level value across all species groupings was as follows: corn 85.7%, soybean 94.5%, wheat 89.8%, cotton 83%, rice 82.5%, and alfalfa 90.7%. For the four aggregated crop groups, the percentages were: i) vegetable and fruits 54.7%, ii) orchard and grapes 49.8%, iii) pasture and hay 93.7%, and iv) others 75.8%

After calculating the Total Applied Toxicity in Equation 1, the eco-efficiency index (*E*) for each crop and species group was determined Eqaution 2 as:


(2) $$E_{s,y,c}=\frac{P_{\mathrm{c,y}}}{TAT_{\mathrm{s,y,c}}}$$

where *P_c,y_* is the total crop production (kg) each year (y) for each crop group and

*TAT_s,y,c_* is the total applied toxicity (kg of active ingredient per toxicity unit or kg/Tu) for species group (s), year (y) and crop group (c). The resulting unit for Eco-efficiency is Tu^−1^. Comparisons across different species groups should be treated with caution since toxicity units differ.

The crop and species-specific eco-efficiency index (*EI*) was calculated by taking the log10 value for each year relative to the corresponding Log10 value for 1992 (**Table 1**) in Equation 3 as:


(3) $$EI_{s,y,c}=\;LogE_{s,y,c}-\;LogE_{s,1992,c}$$

where Log*E_s,y,c_* is the log of eco-efficiency for each species group (s), each year (y) for each crop group (c) and Log*E_s,1992,c_* is the log of eco-efficiency for each species group (s) in 1992 for each crop group (c) (unitless). A log transformation makes a variable unitless (51) and was applied to enhance the interpretability and presentation of the index, making it easier to analyze and visually represent. All data were aggregated, processed, and analyzed using Python 3.12 with the help of NumPy 1.26.4 and Pandas 2.2.3 libraries or using R 4.4.1 and the readxl, reshape2, MASS, gnorm, ggplot2, and latex2exp packages. **Supplementary Figure S1** presents a streamlined overview of the methodological process, offering a simplified breakdown of each step involved.

**Table 1:** Eco-efficiency baseline scores (EI_s,1992,c_) for terrestrial and aquatic species across ten crop categories.

Crop category	Terrestrial				Aquatic		
	Mammals	Birds	Plants	Arthropods	Fish	Invertebrates	Plants
Alfalfa	4.1	3.4	7.4	5	0.6	−1.1	1.4
Corn	5.1	4.3	7.3	6.7	0.7	−0.3	1.1
Cotton	3.4	2.6	6.5	4.2	−0.7	−2.5	0.3
Orchard and grapes	4.7	4	7.8	5.7	0.6	−0.4	1.4
Other	5	4.2	7.4	6.2	0.9	−0.2	1.6
Pasture and hay	5	4.2	6.6	5.8	1.1	−0.3	0.9
Rice	4.6	3.9	7.9	6.4	1.2	0.1	0.8
Soybean	5.4	4.7	7.2	6.4	1.1	0	1
Vegetable and fruits	4.5	3.8	7.5	5.6	0.5	−0.7	1.4
Wheat	5.3	4.9	7	6.1	1.4	0	2
Average	4.7	4.0	7.3	5.8	0.7	−0.5	1.2
Standard deviation	0.6	0.7	0.5	0.7	0.6	0.8	0.5

We analyzed change in *EI*_s,y,c_, from 1992 to 2018 for each crop and species group using an adjusted Theil-Sen estimator. The standard Theil-Sen estimator (52) is typically used when the assumptions of ordinary least squares regression, such as normality, are not met. In our case, the eco-efficiency index is not normally distributed, nor is it expected to be, as it is a transformation of a ratio. However, because the eco-efficiency index calculated above is relative to the baseline year of 1992, the intercept must be zero. Using the standard Theil-Sen estimator without accounting for this constraint would result in biased slope estimates (**Supplementary R Code**).

To address this constraint, we adjusted the calculation by determining the slope between each observed eco-efficiency index, *EI*_s,y,c_, and the origin for a given crop and species group. The median of these slope estimates was then used as the final slope estimate. For example, the eco-efficiency trend for mammals exposed to pesticides from alfalfa crops was calculated by determining the slope of each pair of consecutive annual values *EI*_s,1993,c_, *EI*_s,1994,c_, etc. relative to the origin, and then computing the median of these slopes.

Since the intercept is constrained to zero, standard bootstrap methods for calculating the standard error, confidence intervals, and p-values for the slope such as in Wilcox (53) are not applicable. Instead, we employed the standard error formula for a simple linear regression slope (54) with the least squares estimate replaced by the adjusted Theil-Sen estimate. We calculated the confidence interval and p-values using this standard error, applying a t-distribution with 26 degrees of freedom. Finally, we performed a multiple testing adjustment of the p-values using the Benjamini-Hochberg step-up procedure for controlling the False Discovery Rate (55).

The eco-efficiency index tracks trends in crop sustainability by assessing the balance between productivity and pesticide-related environmental impacts. An increasing index suggests improved sustainability due to higher yields, lower Total Applied Toxicity, or both. Conversely, a declining index indicates inefficiencies, due to reduced productivity, increased pesticide impact, or both. Improved eco-efficiency does not always indicate reduced environmental risks. For example, improved yields due to favorable weather can increase eco-efficiency, even if pesticide use rises, as long as productivity gains offset toxicity increases. This complexity necessitates a careful interpretation of trends within the broader agronomic and environmental context.

This index is designed to measure progress within specific crops rather than facilitating direct comparisons across crops, as pest management challenges and available pesticide options vary significantly. For example, crops with diverse pest complexes, resistant pests, or fewer registered pesticides may exhibit different eco-efficiency patterns than those with less pest pressures and greater management options. Additionally, the index reflects changes in pesticide impact across species groups but does not distinguish between effects on target pests and non-target organisms. For some modern selective insecticides, eco-efficiency may be enhanced by reducing toxicity to beneficial species while maintaining effectiveness against pests. Understanding these nuances is critical for accurately assessing sustainability progress in crop pest management.

## 3 Results

**Figure 1:**
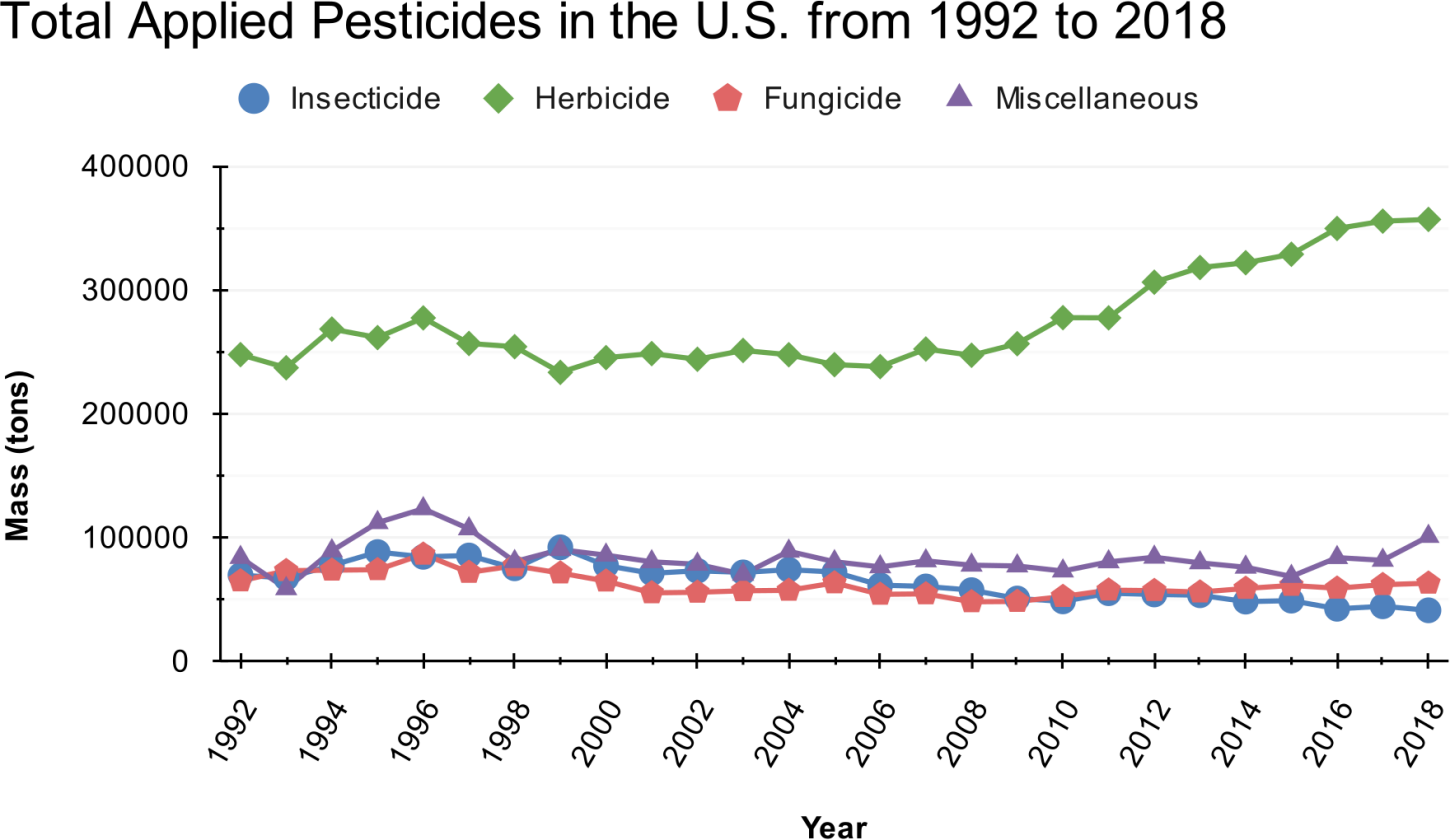
Total mass of applied pesticides by pesticide type in the U.S. from 1992 to 2018.

The total mass of the applied active ingredients in the U.S. steadily increased until peaking at approximately 0.5 million tons in 1996. Afterward, there was a gradual decline until around 2009, followed by a renewed rise, nearly matching the 1996 peak in 2018. Herbicides make up the largest portion of active ingredients applied (**Figure 1**). Herbicides are applied at much higher rates than other types of pesticides, largely driving overall trends in pesticide usage (8). The total mass of herbicides applied in the U.S. experienced a small peak in 1996, followed by a decline until 1999. From 2000 to 2008, herbicide applications exhibited minimal change. However, around 2009, herbicide applications increased until 2018, reaching its overall maximum application mass. In contrast, the total mass of insecticides, fungicides, and other miscellaneous pesticides have remained relatively stable over time. There was a small rise in total applied insecticides beginning in 1993 and peaking in 1999 with a decline thereafter. Total applied fungicides increased from 1992 to 1996 but from 1998 until 2010, total applied fungicides gradually declined and remained relatively stable. The “Miscellaneous” category includes active ingredients of bactericides, fumigants, nematicides, plant growth regulators, and vertebrate control agents.

**Figure 2:**
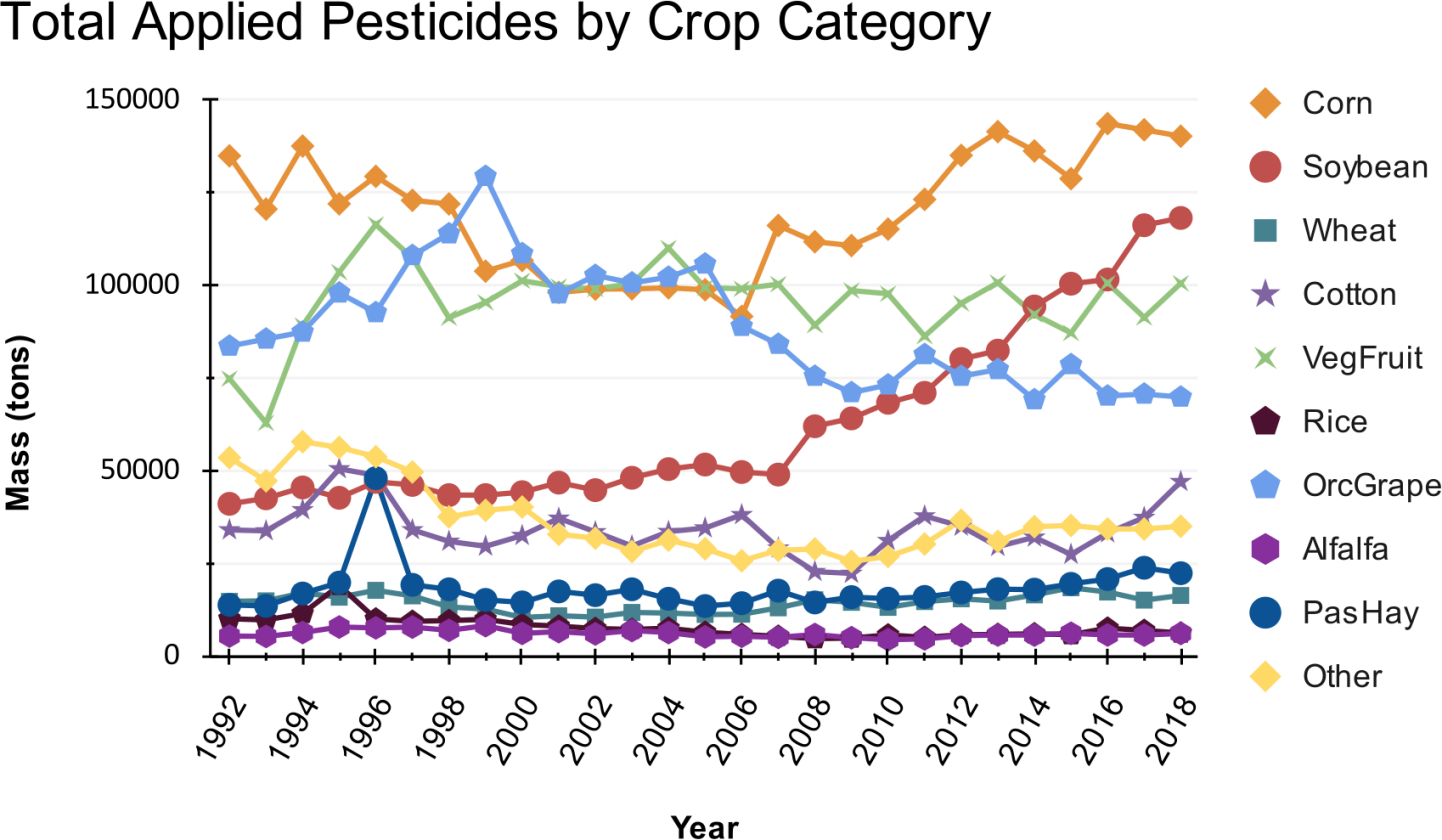
Total mass of applied pesticides by crop category in the U.S. from 1992 to 2018.

When analyzing pesticide use trends across different crop categories, clear and distinct patterns emerged. Corn consistently ranks for the highest pesticide use across all crop groups (**Figure 2**). Pesticide use in soybean has steadily increased since around 2007. In contrast, pesticide use in vegetable and fruits has remained relatively stable overall, though these crops are among the highest users, with significant peaks in 1996 and 2004. Pesticide use in cotton rose until approximately 1995, followed by a decline and subsequent minor fluctuations in the following years. Pesticide use in orchard and grapes experienced a steady increase, peaking in 1999, but have demonstrated a consistent decline since then. Crops such as wheat, alfalfa, pasture and hay, and rice consistently utilize less pesticide compared to corn, soybeans, vegetable and fruits, and orchard and grapes. These variations in the total applied mass of pesticides across crop groups influence eco-efficiency trends.

**Figure 3:**
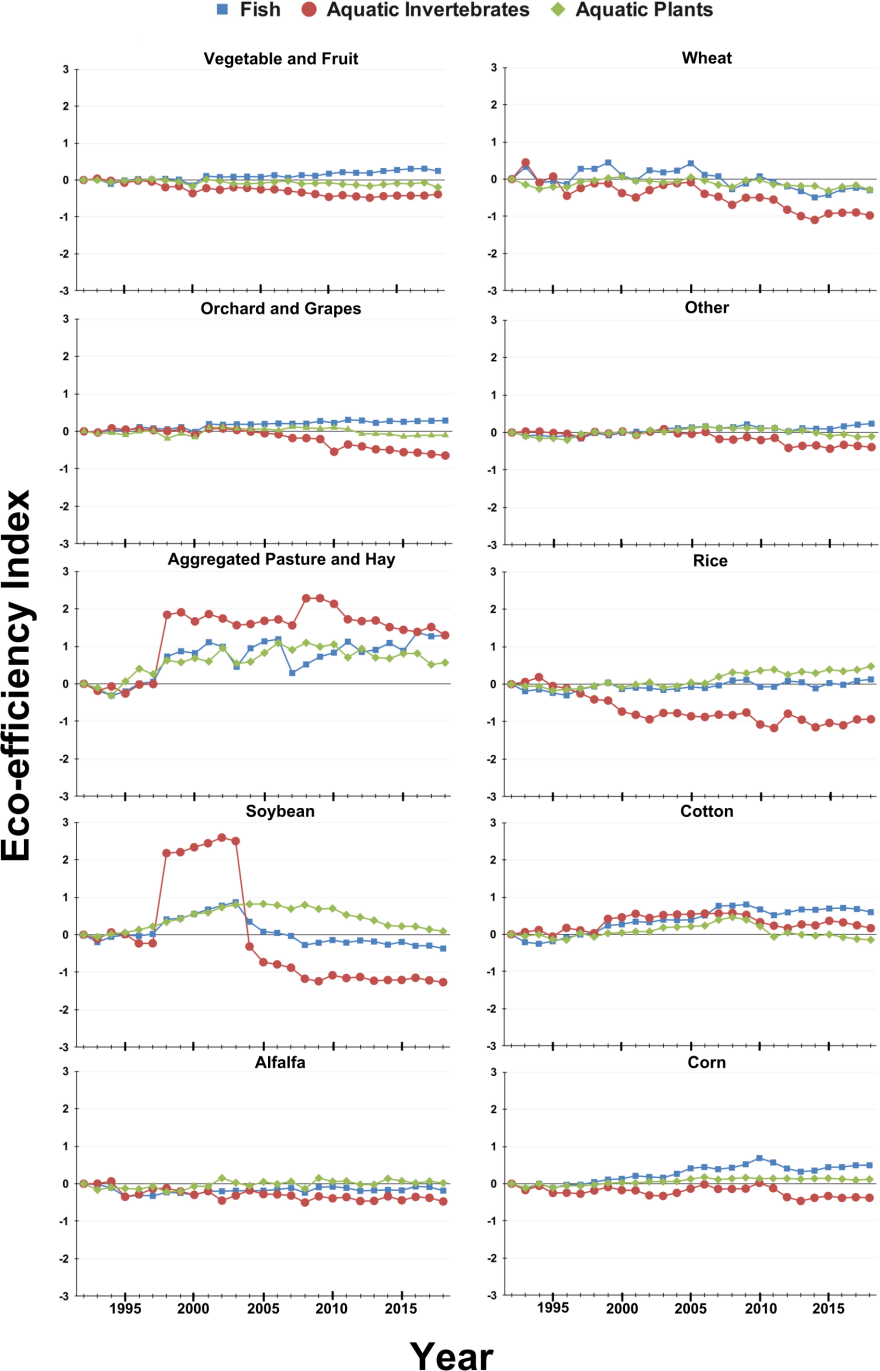
Eco-efficiency indices for 10 crop categories from 1992 to 2018 for aquatic groups. Index values represent crop and species group specific changes in eco-efficiency from 1992 using a log transformation, where positive changes represent an increase in eco-efficiency and negative changes represent a decrease in eco-efficiency.

The eco-efficiency trends for aquatic organisms (**Table 2**, **Figure 3**), including aquatic invertebrates, aquatic plants, and fish, demonstrated a mix of declines, improvements, and neutral changes across crop types. For aquatic invertebrates, steady declines were evident in the plotted trends for alfalfa, corn, rice, and wheat crops (**Figure 3**). The analysis above supports these visual conclusions since the slopes for alfalfa, corn, rice, and wheat crops for aquatic invertebrates were negative and statistically significant (**Table 2**). Additionally, the analysis above revealed a significant negative slope for eco-efficiency in aquatic invertebrates in the vegetable and fruits category (**Table 2**). On the other hand, statistically significant positive slopes were found for cotton and the combined pasture and hay crops for the eco-efficiency indices calculated for aquatic invertebrates (**Table 2**). In the case of aquatic plants, trends in the line plots were largely neutral for most crops with only a visible decline for wheat crops and small improvements for pasture and hay and rice crops. However, the analysis above using the eco-efficiency indices calculated for aquatic plants found significant positive trends for corn and pasture and hay but not rice and significant negative trends for vegetable and fruits for aquatic plants. Note, while there is a statistically significant positive trend in soybean for aquatic plants, the statistical analysis does not account for the non-linear nature of the eco-efficiency index over the years; thus, the actual slope value is likely inflated. For fish, improvements were more widespread; improvements were apparent for corn, cotton, and pasture and hay (**Figure 3**) which were again supported with statistically significant positive slopes. Further, the statistical analysis on the eco-efficiency indices calculated for fish indicated significant positive trends for orchard and grapes, and vegetable and fruits, while neutral trends or minor declines were noted for other crops.

**Table 2:** Slopes of eco-efficiency scores for terrestrial and aquatic species across ten crop categories.

Crop category	Terrestrial				Aquatic		
	Mammals	Birds	Plants	Arthropods	Fish	Invertebrates	Plants
Alfalfa	0.028	0.034	−0.006	0.002	−0.015*	−0.021*	−0.001
Corn	0.037*	0.049*	−0.012*	−0.02	0.019*	−0.018*	0.005*
Cotton	0.042*	0.033*	−0.009	0.03*	0.03*	0.033*	0.003
Orchard and grapes	0.031*	0.046*	−0.013*	0.01*	0.013*	−0.012	−0.002
Other	0.045*	0.024*	0.005	0.014	0.005	−0.007	0.001
Pasture and hay	0.024*	0.064*	−0.009	0.108*	0.048*	0.097*	0.051*
Rice	0.054*	0.09*	−0.008	−0.026*	−0.005	−0.052*	0.005
Soybean	0.022	0.036	−0.003	−0.024	−0.008	−0.051	0.04*
Vegetable and fruits	0.028*	0.014	−0.011*	0	0.008*	−0.02*	−0.005*
Wheat	0.007	0.025*	−0.021*	0.003	−0.004	−0.034*	−0.008
Average	0.032	0.042	−0.009	0.010	0.009	−0.008	0.009

Statistical significance with Benjamini-Hochberg correction *P<0.05.

**Figure 4:**
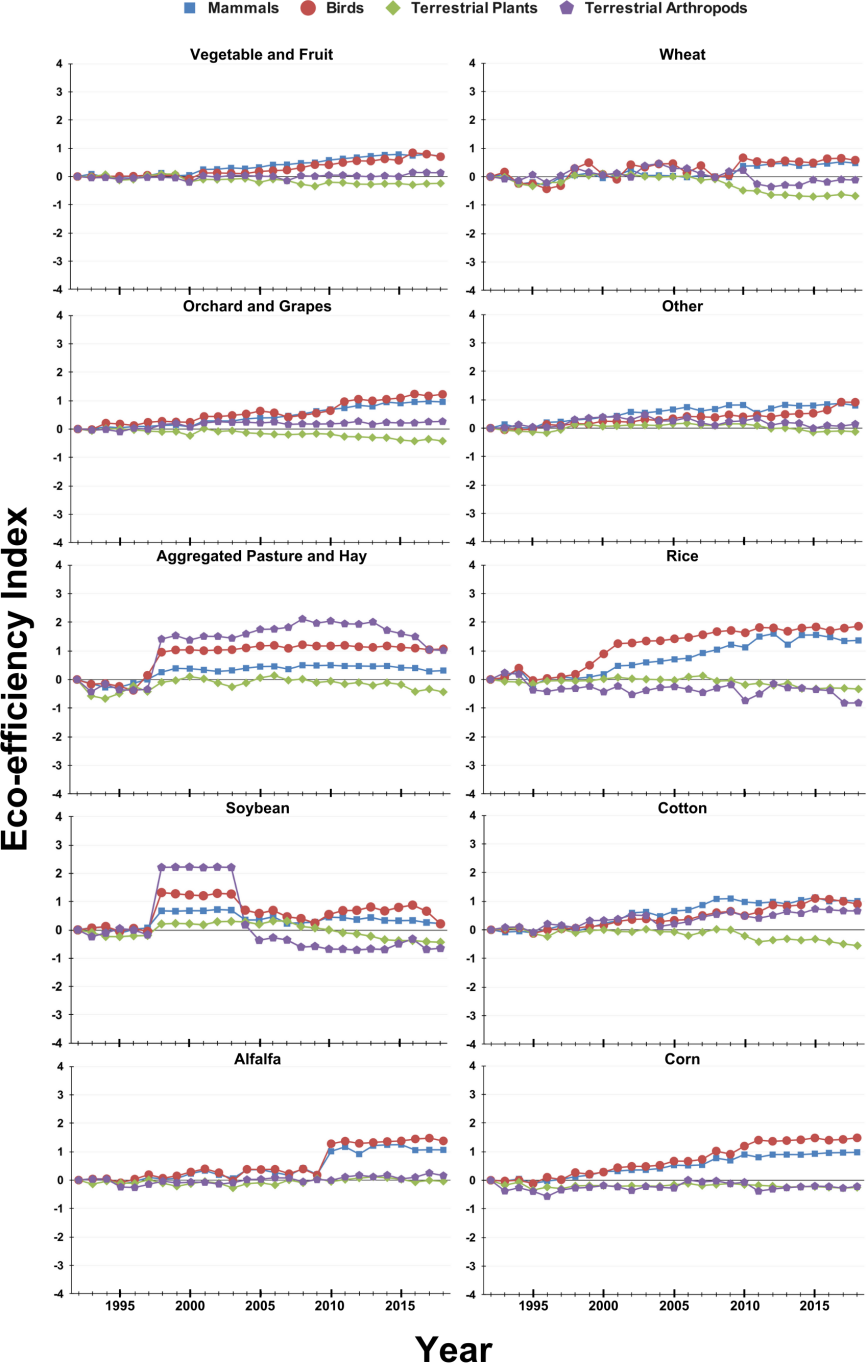
Eco-efficiency indices for 10 crop categories from 1992 to 2018 for terrestrial groups.

The eco-efficiency trends for terrestrial organisms (**Table 2**), including birds, mammals, terrestrial arthropods, and terrestrial plants, revealed varied outcomes across crops. Improvements were widespread for birds and mammals, with visible positive steady trends observed in crops like alfalfa, corn, cotton, orchard and grapes, the combined other crop, and rice (**Figure 4**). Again, the results of the analysis agreed with these visible trends, with significant positive slopes for all specie groups except alfalfa (**Table 2**). Additionally, the slopes for the eco-efficiency index for mammals and birds were significantly positive for vegetable and fruits as well as for birds in wheat eco-efficiency (**Table 2**). Finally, while the analysis suggested significant positive trends for mammals and birds in pasture and hay, the slopes are likely inflated due to the exogenous shock observed around 1998 (**Table 2**, **Figure 4**). However, trends for terrestrial arthropods were mixed with crops such as cotton and orchard and grapes showing improvement, while rice had a visible decline (**Figure 4**). The statistical analysis on the eco-efficiency indices calculated for terrestrial arthropods confirmed that there are statistically significant positive trends for corn and cotton and a significant negative trend for rice (**Table 2**). Again, while a significant positive trend was found for pasture and hay, the analysis does not account for non-linear trends or exogenous shocks, and thus these trends are likely inflated (**Table 2**, **Figure 4**). Finally, the eco-efficiency index appeared to remain stable or declined for terrestrial plants (**Figure 4**). In fact, significant declines of the eco-efficiency index for terrestrial plants were found for corn, orchard and grapes, vegetable and fruits, and wheat (**Table 2**). However, it is important to note that some of these declines began around 2010, meaning the slopes calculated using the method above may not accurately capture the true trends (**Table 2**, **Figure 4**).

The eco-efficiency trends observed across crop categories (**Table 2**) indicate notable variation. Pasture and hay exhibited the most consistent improvement across all species groups. Cotton and orchard and grapes also demonstrated improvements for multiple species. In contrast, crops such as wheat and vegetable and fruits showed declines or no significant changes across several species categories. Corn presented mixed results, with significant improvements for mammals and birds but declines for terrestrial arthropods and aquatic invertebrates. Rice displayed improvement for certain specie groups but declines in others.

## 4 Discussion

Pesticide use decisions are influenced by a dynamic set of factors that shape pest management strategies. Weather conditions, such as temperature, precipitation, and extreme weather events, play a significant role in determining pest activity and the need for pesticide applications. Climate change has increasingly shaped weather patterns, allowing insect pests to expand their ranges, survive winters more effectively, and reproduce at higher rates, all of which intensify pest pressures and pose greater challenges for agriculture and ecosystem stability (56).

While environmental factors play a crucial role in pest management, this discussion focuses on broader, systemic influences such as regulatory changes, market dynamics, and the evolution of pest pressures and resistance. These interconnected forces have shaped pest management strategies, driving decisions on pesticide selection, technological advancements, and the adoption of IPM practices (8, 57). The variation in potential downstream effects on non-target organisms, as reflected in the eco-efficiency indices evaluated in this study, highlights the value of tools like eco-efficiency in analyzing and monitoring these dynamic changes over time, providing essential insights into pesticide use patterns and their broader implications for environmental sustainability and public health. We do not claim a causal relationship between the factors examined here and changes in eco-efficiency. Instead, our focus is on understanding how various influences on pesticide use decision-making may contribute to shifts in eco-efficiency.

Concerns about pesticides intensified among consumers in the 1990s after the discovery of pesticide residues in food, especially in products frequently consumed by children, prompting calls for regulatory reform (58). The Food Quality Protection Act of 1996 marked a key shift, introducing stricter EPA safety standards, particularly for children, and leading to reduced use of high-rate, toxic pesticides like carbamates and organophosphates that have been largely replaced by alternatives like neonicotinoids and pyrethroids with lower application rates and toxicity to mammals, birds, and fish (8, 57). Although neonicotinoids and pyrethroids have reduced toxicity to vertebrates and lowered the total pesticide mass applied, their widespread use has dramatically increased toxicity to insects (9, 59). While regulatory reforms have successfully reduced pesticide risks to humans and other vertebrates, the shift toward widespread use of neonicotinoids highlights the unintended ecological trade-offs, underscoring the need for a more holistic approach to pesticide regulation and risk assessment.

Beyond domestic regulatory changes, international regulations on pesticide residues have become increasingly stringent, aiming to mitigate potential risks to consumers and enhance food safety standards worldwide (60). The U.S. has one of the largest export markets in the world, with agricultural goods playing a vital role in driving economic growth (61). U.S. agricultural exports are increasingly affected by Maximum Residue Levels, which are set by individual countries to regulate the allowable pesticide concentrations in agricultural products (62). These varying standards create challenges for exporters, requiring careful compliance with international regulations to maintain market access, directly shaping pesticide use decisions made by growers (63). These international restrictions have the potential to accelerate both domestic and global advancements in eco-efficiency by encouraging innovation in pest management strategies.

In addition to policy changes, market dynamics have played a pivotal role in shaping eco-efficiency in agriculture, driven by the balance between input costs, such as pesticides, and crop parity price (8). The parity ratio, which reflects the relationship between farm prices and input costs, showed an overall decline during the study period, reaching an all-time low in 2018 (64). When production costs outpace crop parity or commodity prices fall, farmers face shrinking profit margins. This market system often drives increased reliance on chemical controls, as pesticides typically have lower upfront costs compared to other more sustainable management options while offering the potential for higher yields in the short term (65). This forces growers to heavily rely on chemical controls for pest management to maintain their market edge. This is especially common with high-value crops, which often require increased pesticide use to protect yields (66). Collectively, these market dynamics are expected to diminish eco-efficiency due to the increased pressure to use pesticides.

Genetically engineered (GE) crops have significantly shaped pesticide use patterns and their effects on eco-efficiency. The introduction of herbicide-tolerant crops in the mid-1990s, particularly glyphosate-tolerant cotton, soybean, and corn, revolutionized weed management. By 2014, these genetically modified varieties accounted for over 90% of total crop area (67). This widespread adoption led to a significant increase in herbicide use, with soybeans experiencing the highest application rates (57). Compared to other herbicides, glyphosate is considered to have lower toxicity and environmental persistence, potentially reducing the overall ecological footprint of pesticide use in these systems (8). However, the overreliance on glyphosate has driven the emergence of glyphosate-resistant weed species, such as Palmer amaranth (*Amaranthus palmeri* S. Watson), forcing growers to shift toward more frequent applications and herbicides with greater toxicity, ultimately diminishing eco-efficiency (9, 68, 69). However, glyphosate is not the only herbicide facing reduced efficacy. Weed species have developed resistance to nearly all existing herbicide classes, and few new modes of action have been introduced in the past 30 years (70, 71). The evolution of herbicide resistance underscores the need for comprehensive assessment tools like eco-efficiency, which can provide a more holistic evaluation of the long-term environmental trade-offs associated with these management decisions.

A closer examination of genetically engineered crops reveals that insect-resistant varieties, particularly *Baccillus thuringiensis* Berlin (*Bt)* crops, exhibit a dual-edged effect similar to herbicide-tolerant crops. Initially, *Bt* corn and cotton significantly reduced conventional insecticide applications, lowering exposure risks for non-target organisms and improving environmental outcomes (57, 72, 73). This decline in total applied insecticides enhances eco-efficiency by reducing the overall pesticide load. However, despite this reduction, the widespread adoption of neonicotinoids and pyrethroids became a victim of their own success; their increased use led to a rise in Total Applied Toxicity for invertebrates (9). In fact, when compared to non-*Bt* crops, the applied toxicity has not decreased for invertebrates as expected, likely due to the development of pest resistance that necessitates increased applications (74). The combination of pesticide class shifts and the evolution of pest resistance has ultimately contributed to an increased toxicity load for invertebrates, even in *Bt* crop systems, highlighting the unintended ecological consequences of these management strategies and their negative implications on eco-efficiency.

Beyond herbicide tolerant and insect resistant crop production systems, resistance to applied insecticides, documented in over 550 arthropod species as of 2018 (71, 75), continues to rise and further complicates long term pesticide management, undermining eco-efficiency progress. Pest resistance reduces the effectiveness of pesticides and increases the overall mass of applied pesticides and toxicity (76). Estimating this additional mass applied in response to resistance is difficult to accomplish. A widely cited figure is that 10% of pesticide applications are applied due to the development of resistance (7). Some caution is needed in the interpretation of this figure as it is based on a simulation study for a single insect species in cotton (77). Pesticide resistance is estimated to cost U.S. agriculture $10 billion in losses annually (71, 78). These losses are expected to rise as pesticide dependence increases and resistance continues to evolve, underscoring the urgent need for effective management strategies.

Although this discussion has heavily focused on insecticide and herbicide resistance, fungicide resistance as a response to routine fungicide use is also a growing concern in many crops, especially for vegetables, fruits, orchard, and grapes (79–82). Similarly, fungicide use in soybean has dramatically risen since 2005 likely due to increased awareness of fungal diseases, fungicide product availability, and crop prices (83). Overall, pest prevalence and resistance are likely to result in an increased mass of applied pesticides and toxicity load resulting in reduced eco-efficiency.

Building on the advancements in GE crops, precision agriculture and related innovations are transforming modern production systems. By leveraging cutting-edge technologies such as robotic sprayers and crop imaging systems, precision agriculture optimizes pesticide applications based on factors like pest pressure and crop canopy density. This targeted approach minimizes pesticide use while enhancing long-term pest management and maintaining pesticide efficacy (84–86). Robotic weeders further enhance efficiency by integrating imaging technologies combined with mechanical, electrical, laser, thermal, or chemical tools for selective weed control, particularly benefitting herbicide management (87). Similarly, unmanned aerial vehicles (UAVs) enable targeted applications of pesticides, further reducing overall pesticide use (88). Beyond machine-based innovations, the development of non-chemical pesticide alternatives, including bio-pesticides (89) and emerging biotechnologies such as interference RNAs (iRNAs) (90), holds significant promise for improving eco-efficiency. Collectively, these technological advancements are expected to pave the way for more sustainable agricultural practices, reducing pesticide volumes and mitigating their environmental impact.

Technological advancements and holistic pest management practices have been widely promoted by regional IPM centers, established in 2000 as part of the mandates outlined in the FQPA of 1996 (8). Common IPM practices, such as crop rotation, host plant resistance, pest monitoring, and threshold-based interventions (26, 32) can improve eco-efficiency by reducing the frequency of pesticide applications and promoting the use of lower-risk pesticides. A compelling example of IPM’s success is Arizona’s cotton production system. By strategically integrating *Bt* cotton, selective insecticides, insect growth regulators, established sampling protocols, and effective economic thresholds, cotton growers have successfully managed pest pressures while simultaneously preserving natural insect communities that contribute to biological control (91). This system has been extensively studied for over thirty years and widely adopted across the region, highlighting key factors that have contributed to its success.

Areawide IPM programs play a crucial role in regional pest suppression by managing pest populations across large areas, rather than isolated fields. This coordinated approach is particularly effective for highly mobile pests, preventing their movement between untreated areas and reducing overall infestation levels. The U.S. Boll Weevil Eradication Program (1998–2000), is a classic example demonstrating the success of areawide IPM programs (92). This collaborative effort among researchers, government agencies, and cotton producers led to the widespread adoption of *Bt* cotton and the targeted use of malathion to control boll weevil (*Anthonomus grandis* Boheman). Other notable examples of area wide pest management include Asian citrus psyllid (*Diaphorina citri* Kuwayama) a vector of citrus greening disease (*“Candidatus Liberibacter”* spp.) (93), and a program for managing glassy-winged sharpshooter (*Homalodisca vitripennis* [Germar]), a vector of Pierce’s disease (*Xylella fastidiosa* Wells et al.) (94). These programs highlight the effectiveness of implementing integrated control strategies on a larger scale to enhance pest management, leading to improved regional management. This approach is expected to positively impact eco-efficiency by minimizing pesticide resistance and promoting IPM strategies.

Overall, sustainability in crop pest management is shaped by a complex interplay of pesticide regulations, market dynamics, and pest pressures. Certain factors, such as GE crops, can have both positive and negative impacts, demonstrating improvements in some cases while leading to unintended challenges in others. The most effective path to sustainability lies in an integrated approach to pest management, recognizing that no single solution serves as a universal fix. This underscores the critical need for ongoing research and Extension efforts to advance innovative pest management technologies and strategies that address evolving challenges. However, these efforts can only succeed with continued support and funding, ensuring that sustainable solutions remain a priority in agricultural systems.

The eco-efficiency index developed in this study provides a more holistic and practical framework for tracking agricultural sustainability compared to traditional methods. Unlike approaches that focus solely on the volume of pesticides applied, this index incorporates a toxicity component, enabling a more nuanced evaluation of environmental impacts. By integrating toxicity values across multiple species groups, the index delivers a comprehensive assessment of the ecological effects of pesticide use, ensuring the diverse impacts on ecosystems are thoroughly considered. While surveys of IPM practices (28, 95) offer valuable insights into sustainable pest management strategies, they do not directly quantify pesticide-related environmental externalities. The eco-efficiency index addresses this gap by providing metrics that capture these externalities, offering a robust tool for evaluating sustainability. Moreover, the index’s user-friendly design enhances its accessibility to a wide range of stakeholders, including policymakers, researchers, and agricultural practitioners. This accessibility ensures that the tool can support informed decision-making and drive advancements in sustainable agricultural practices.

A key strength of the eco-efficiency index is its integration of crop production data, enabling the assessment of sustainability improvements independent of production changes. This ensures that reductions in pesticide use reflect genuine advancements in management practices rather than declines in agricultural output. Additionally, the use of Toxicity Level values provides a standardized, scientific benchmark for interpreting pesticide toxicity (9, 42) with greater complexity than simpler metrics such as the EU’s Harmonised Risk Indicators (96). This approach enhances the reliability and comparability of eco-efficiency assessments across various crops and regions, fostering data-driven decision-making. The adoption and measurement of eco-efficiency scores in U.S. crops could address a critical limitation in measuring IPM progress and impacts (24). Specifically, the eco-efficiency index has the potential to serve as a valuable metric within broader sustainability frameworks. For instance, a widely recognized agricultural sustainability system currently evaluates climate, soil, energy, water, and land use for major crops but omits pesticide use (97). Integrating an eco-efficiency index into such systems would fill this gap, offering a more comprehensive evaluation of agricultural sustainability.

A notable limitation of this analysis is its dependence on relatively coarse production and pesticide use data sourced from NASS and USGS. This is a common challenge in eco-informatic studies that rely on large datasets from multiple sources, which may lack uniformity and completeness (98). While these datasets offer valuable insights into national trends, their temporal and spatial resolutions are insufficiently detailed to capture localized or crop-specific variations in pesticide application. This lack of detail may obscure critical differences in pest pressures, management practices, and environmental conditions across regions or even individual fields. The limited accuracy of pesticide data has been recognized as a constraint for regulatory decision-making, particularly in the context of the EPA’s Endangered Species Act (ESA) implementation (99). Additionally, the broad crop groupings used in this study, though necessary for data aggregation, further mask variability among specific crops within each category. For instance, grouping vegetable and fruits combines a wide array of crops with distinct pesticide use profiles, potentially concealing crop-specific eco-efficiency trends. Addressing these limitations in future studies would enhance the precision and applicability of eco-efficiency assessments.

Another limitation of this study is the incomplete availability of Toxicity Level values for all active ingredients. Toxicity Levels are essential for assessing pesticide toxicity and environmental impact, but data gaps for certain pesticides require approximations or exclusions, thereby reducing the precision of the eco-efficiency estimates. While our analysis focused on terrestrial arthropods, it did not specifically examine pollinators due to a lack of Toxicity Level data. However, this could be an area of focus for future studies. Notably, a recent study investigated the toxicity of pesticides on pollinators in corn and soybean systems, incorporating an eco-efficiency analysis to evaluate the environmental impact (100).

To address these limitations, future efforts should prioritize the development of Toxicity Levels for a broader range of pesticides to enhance the robustness of eco-efficiency calculations. Creating a publicly accessible database of Toxicity Levels would also promote transparency and facilitate the wider application of eco-efficiency indices. While the Toxicity Levels used in this study are based on official U.S. EPA databases, the agency has not established Regulatory Toxicity Levels for agricultural pesticide use. Although the eco-efficiency index developed here can be used independently of such endorsements, input from the EPA would add significant value and credibility to the process, further improving the utility and acceptance of the index.

A notable omission in this analysis is the lack of consideration for pesticide persistence and exposure in the environment. While toxicity is a critical factor in assessing pesticide impact, persistence (the duration a pesticide remains active in the environment) and exposure (the frequency and extent to which non-target species, including humans, encounter the pesticide) are equally important for evaluating environmental and human health risks. Research has shown that Toxicity Level values are valid for assessing toxicological risks under varying degrees of exposure refinement (44). The mass of applied pesticides is a simplistic form of exposure level and could be refined in future studies. Exposure risks, particularly for agricultural workers, have been substantially reduced over the period of this study through advancements such as enclosed tractor cabins with filtration systems, enclosed mixing systems and standards to protect agricultural workers (101). Similarly, agricultural producers now widely adopt drift and runoff mitigation practices, including vegetation buffer strips, drift-reducing adjuvants, swath control, and improved runoff management strategies. Compliance with EPA ESA regulations often necessitates that producers adopt specific mitigations with a point-based system if they are in Pesticide Use Limitation Areas (102). However, persistent pesticides with low acute toxicity can accumulate in ecosystems, causing long-term environmental harm, while highly toxic pesticides applied in localized areas may result in minimal exposure to non-target species (103, 104). Incorporating persistence and exposure into the eco-efficiency index would provide a more comprehensive evaluation of pesticide impacts and align the analysis more closely with real-world conditions, enhancing its utility for sustainability assessments. Future iterations of the index could incorporate other factors impacting pesticide use including land use (97) as well as carbon and energy use (105).

The simplified index developed in this study has several limitations but serves as a valuable proof of concept, demonstrating the potential of eco-efficiency metrics to evaluate the environmental impacts of pesticide use across major crops and species groups. By utilizing publicly available datasets and incorporating species-specific metrics, this study establishes a framework for analyzing broad eco-efficiency trends and identifying opportunities for targeted improvements. The findings align with established research, highlighting the positive effects of genetically engineered crops and IPM practices. At the same time, they underscore persistent challenges, such as herbicide resistance and the externalities of pesticide use, including runoff and drift. These issues are increasingly scrutinized under the EPA’s ESA implementation reflecting their critical role in shaping modern pesticide regulations (106). Another limitation is the potential for incentive misalignment, wherein improvements to the Eco-efficiency Index may be achieved through superficial or administrative changes, such as switching to pesticides with lower toxicity but that are more persistent in the environment, without delivering meaningful reductions in overall environmental impact.

As noted earlier, there are limitations to the statistical analysis used to quantify trends in the eco-efficiency indices of different lifeforms by crop. The primary limitation is that the analysis assumes linear trends, while some data exhibit nonlinear patterns or exogenous shocks. Another key limitation lies in the estimation of the standard error, which is used to assess the significance of the slopes. Standard bootstrap methods could not be applied due to the constraint that the regression intercept must be zero. Instead, a modified version of the standard error estimation for ordinary least squares regression was employed. However, the impact of this modification on the accuracy of the standard error estimate remains unclear. Additional research is required to evaluate the effects of this approach and to develop improved methods for estimating the standard error under these specific constraints.

## 5 Conclusion

This study highlights the potential use of the eco-efficiency indicator presented in this work as a tool for assessing the environmental impacts of pesticide use. By refining the data sources and expanding the scope to include persistence, exposure, and additional crop-specific details, future research can build on this framework to provide more nuanced and actionable insights. Expanding the scope to include specific pesticide active ingredients used in genetically modified crops and addressing data gaps in Toxicity Level values would also enhance its applicability and precision. These improvements will be essential for guiding policy decisions, prioritizing research and extension efforts, optimizing pest management strategies, and advancing sustainable agriculture. Such an effort would require a multidisciplinary team and a diverse group of stakeholders to successfully develop the index. One valuable forum for stakeholder engagement is the Pest Management Strategic Plan workshops, which brings together growers, consultants, Extension professionals, and regulators (107). These workshops comprehensively review all aspects of pest management of a specific crop and region. To promote broader adoption of IPM, we recommend incorporating eco-efficiency indices and performance targets as practical tools to inform pesticide decision-making, encourage the judicious and selective use of pesticides, and benchmark progress toward risk reduction goals.

## Data Availability

The original contributions presented in the study are included in the article/**supplementary material**. Further inquiries can be directed to the corresponding author.

## References

[1] Bonner MR Alavanja MC. Pesticides, human health, and food security. Food Energy Secur. (2017) 6(3):89–93. doi: 10.1002/fes3.112

[2] Popp J Pető K Nagy J. Pesticide productivity and food security. A review. Agron Sustain Dev. (2013) 33:243–55. doi: 10.1007/s13593-012-0105-x

[3] Weller S Culbreath A Gianessi L Godfrey L Jachetta J Norsworthy J. The contributions of pesticides to pest management in meeting the global need for food production by 2050. Issue Paper-Council Agric Sci Technol. (2014) 55. https://www.ag.ndsu.edu/potatoextension/the-contributions-of-pesticides-to-pest-management-in-meeting-the-global-need-for-food-production-by-2050.

[4] Maggi F Tang FH Tubiello FN. Agricultural pesticide land budget and river discharge to oceans. Nature. (2023) 620:1013–7. doi: 10.1038/s41586-023-06296-x 37438527

[5] Tang FH Lenzen M McBratney A Maggi F. Risk of pesticide pollution at the global scale. Nat Geosci. (2021) 14:206–10. doi: 10.1038/s41561-021-00712-5

[6] Bourguet D Guillemaud T. The hidden and external costs of pesticide use. In: Lichtfouse E, editor. Sustainable agriculture reviews. Springer, Switzerland (2016). p. 35–120.

[7] Pimentel D Burgess M. Environmental and economic costs of the application of pesticides primarily in the United States. In: Pimentel RPD, editor. Integrated pest management. Dordrecht, Netherlands: Springer (2014). p. 47–71.

[8] Fernandez-Cornejo J Nehring R Osteen C Wechsler S Martin A Vialou A. Pesticide use in US agriculture: 21 selected crop 2006-2008. Washington, D.C., U.S.: USDA ERS Economic Information Bulletin Number 124 (2014).

[9] Schulz R Bub S Petschick LL Stehle S Wolfram J. Applied pesticide toxicity shifts toward plants and invertebrates, even in GM crops. Science. (2021) 372:81–4. doi: 10.1126/science.abe1148 33795455

[10] Khalifa SAM Elshafiey EH Shetaia AA El-Wahed AAA Algethami AF Musharraf SG. Overview of bee pollination and its economic value for crop production. Insects. (2021) 12:688. doi: 10.3390/insects12080688 34442255 PMC8396518

[11] Klatt BK Holzschuh A Westphal C Clough Y Smit I Pawelzik E. Bee pollination improves crop quality, shelf life and commercial value. Proc R Soc B: Biol Sci. (2014) 281:20132440. doi: 10.1098/rspb.2013.2440 PMC3866401 24307669

[12] Reilly JR Artz DR Biddinger D Bobiwash K Boyle NK Brittain C. Crop production in the USA is frequently limited by a lack of pollinators. Proc R Soc B: Biol Sci. (2020) 287:20200922. doi: 10.1098/rspb.2020.0922 PMC7423660 33043867

[13] Ara ZG Haque AR. A comprehensive review on synthetic insecticides: toxicity to pollinators, associated risk to food security, and management approaches. J Biosyst Eng. (2021) 46:254–72. doi: 10.1007/s42853-021-00104-y

[14] Goulson D Nicholls E Botías C Rotheray EL. Bee declines driven by combined stress from parasites, pesticides, and lack of flowers. Science. (2015) 347:1255957. doi: 10.1126/science.1255957 25721506

[15] Hallmann CA Sorg M Jongejans E Siepel H Hofland N Schwan H. More than 75 percent decline over 27 years in total flying insect biomass in protected areas. PloS One. (2017) 12:e0185809. doi: 10.1371/journal.pone.0185809 29045418 PMC5646769

[16] Sánchez-Bayo F Wyckhuys KAG. Worldwide decline of the entomofauna: A review of its drivers. Biological Conservation. (2019) 232:8–27. doi: 10.1016/j.biocon.2019.01.020

[17] Vanbergen AJ. Threats to an ecosystem service: Pressures on pollinators. Front Ecol Environ. (2013) 11:251–9. doi: 10.1890/120126

[18] Wagner DL Grames EM Forister ML Berenbaum MR Stopak D. Insect decline in the Anthropocene: Death by a thousand cuts. Proc Natl Acad Sci. (2021) 118:e2023989118. doi: 10.1073/pnas.2023989118 33431573 PMC7812858

[19] Knopper LD Dan T Reisig DD Johnson JD Bowers LM. Sugar concentration in nectar: A quantitative metric of crop attractiveness for refined pollinator risk assessments. Pest Manage Sci. (2016) 72:1807–12. doi: 10.1002/ps.4321 PMC5094517 27197566

[20] Sgolastra F Medrzycki P Bortolotti L Maini S Porrini C Simon-Delso N. Bees and pesticide regulation: Lessons from the neonicotinoid experience. Biol Conserv. (2020) 241:108356. doi: 10.1016/j.biocon.2019.108356

[21] Shahmohamadloo RS Tissier ML Guzman LM. Risk assessments underestimate threat of pesticides to wild bees. Conserv Lett. (2024) 17:e13022. doi: 10.1111/conl.13022

[22] Uhl P Brühl CA. The impact of pesticides on flower-visiting insects: A review with regard to european risk assessment. Environ Toxicol Chem. (2019) 38:2355–70. doi: 10.1002/etc.4572 31408220

[23] Weisner O Frische T Liebmann L Reemtsma T Roß-Nickoll M Schäfer RB. Risk from pesticide mixtures – The gap between risk assessment and reality. Sci Total Environ. (2021) 796:149017. doi: 10.1016/j.scitotenv.2021.149017 34328899

[24] United States General Accounting Office (GAO). Agricultural pesticides: management improvements needed to further promote integrated pest management. In: US GAO GAO-01-815. Washington, DC: United States General Accounting Office (GAO) (2001).

[25] USDA. U.S. Department of Agriculture. 2018. National road map for Integrated Pest Management, Revised September 21, 2018. Washington, D.C.: U.S. Department of Agriculture. (2018).

[26] Dara SK. The new integrated pest management paradigm for the modern age. In J Integrated Pest Manage (Vol. 10 Issue 1. (2019) p:12). doi: 10.1093/jipm/pmz010

[27] Jacobsen B. USDA integrated pest management initiative. In: Radcliffe EB Hutchison WD Cancelado RE, editors. Radcliffe’s IPM world textbook. University of Minnesota, St. Paul, MN (1996). Available at: https://ipmworld.umn.edu.

[28] Farrar JJ. Using National Agricultural Statistics Service pest management practices survey to assess IPM adoption in California. J Integrated Pest Manage. (2023) 14:24. doi: 10.1093/jipm/pmad022

[29] Brewer MJ Goodell PB. Approaches and incentives to implement integrated pest management that addresses regional and environmental issues. Annu Rev Entomology. (2012) 57:41–59. doi: 10.1146/annurev-ento-120709-144748 21888519

[30] Lane DE Walker TJ Grantham DG. IPM adoption and impacts in the United States. J Integrated Pest Manage. (2023) 14:1. doi: 10.1093/jipm/pmac028

[31] Greenway GA Asiseh F Quaicoe O. A cost benefit analysis of IPM decision support tools for potato psyllids in idaho, oregon, and washington. Am J Potato Res. (2021) 98:122–9. doi: 10.1007/s12230-021-09823-6

[32] Pecenka JR Ingwell LL Foster RE Krupke CH Kaplan I. IPM reduces insecticide applications by 95% while maintaining or enhancing crop yields through wild pollinator conservation. Proc Natl Acad Sci. (2021) 118:e2108429118. doi: 10.1073/pnas.2108429118 34697238 PMC8612243

[33] Bourguet D Guillemaud T. The hidden and external costs of pesticide use. In: Sustainable agriculture reviews. Springer, Cham (2016). p. 35–120. doi: 10.1007/978-3-319-26777-7_2

[34] Pimentel D Hepperly P Hanson J Douds D Seidel R. Environmental, energetic, and economic comparisons of organic and conventional farming systems. Bioscience. (2005) 55:573–82. doi: 10.1641/0006-3568(2005)055[0573:EEAECO]2.0.CO;2

[35] EC. Communication From The Commission To The European Parliament, The Council, The European Economic And Social Committee And The Committee Of The Regions—Chemicals Strategy For Sustainability, Towards A Toxic-Free Environment. Brussels, 14.10.2020 COM(2020) 667 final (2020). Available online at: https://ec.europa.eu/environment/pdf/chemicals/2020/10/Strategy.pdf (Accessed November 1, 2024).

[36] Silva V Yang X Fleskens L Ritsema CJ Geissen V. Environmental and human health at risk–Scenarios to achieve the Farm to Fork 50% pesticide reduction goals. Environ Int. (2022) 107296. doi: 10.1016/j.envint.2022.107296 35580470

[37] Burdeau C. Bowing to farmers, EU scraps pesticide rule, shields farming from tough 2040 emissions cuts. Courthouse News Service (2024). Available at: https://www.courthousenews.com/bowing-to-farmers-eu-scraps-pesticide-rule-shields-farming-from-tough-2040-emissions-cuts/ (Accessed November 1, 2024).

[38] Clancy MS Moschini G. Mandates and the incentive for environmental innovation. Am J Agric Economics. (2018) 100:198–219. doi: 10.1093/ajae/aax051

[39] Magarey RD Klammer SS Chappell TM Trexler CM Pallipparambil GR Hain EF. Eco-efficiency as a strategy for optimizing the sustainability of pest management. Pest Manage Sci. (2019) 75:3129–34. doi: 10.1002/ps.5560 31318146

[40] Acosta-Alba I Chia E Andrieu N. The LCA4CSA framework: Using life cycle assessment to strengthen environmental sustainability analysis of climate smart agriculture options at farm and crop system levels. Agric Syst. (2019) 171:155–70. doi: 10.1016/j.agsy.2019.02.001

[41] USDA. Climate-smart agriculture and forestry strategy: 90-day progress report. Washington, D.C.: U.S. Department of Agriculture (2020).

[42] Stehle S Schulz R. Agricultural insecticides threaten surface waters at the global scale. Proc Natl Acad Sci. (2015) 112:5750–5. doi: 10.1073/pnas.1500232112 PMC4426442 25870271

[43] Hamilton DJ Ambrus Á Dieterle RM Felsot AS Harris CA Holland PT. Regulatory limits for pesticide residues in water (IUPAC Technical Report). In Pure Appl Chem. (2003) 75:1123–1155). doi: 10.1351/pac200375081123

[44] Peterson RK. Comparing ecological risks of pesticides: The utility of a risk quotient ranking approach across refinements of exposure. Pest Manage Sci. (2006) 62:46–56. doi: 10.1002/ps.v62:1 16217731

[45] Kniss AR Coburn CW. Quantitative evaluation of the environmental impact quotient (EIQ) for comparing herbicides. PloS One. (2015) 10:e0131200. doi: 10.1371/journal.pone.0131200 26121252 PMC4487257

[46] Peterson RK Schleier JJ III. A probabilistic analysis reveals fundamental limitations with the environmental impact quotient and similar systems for rating pesticide risks. PeerJ. (2014) 2:e364. doi: 10.7717/peerj.364 24795854 PMC4006226

[47] Maggi F Tang FH la Cecilia D McBratney A. PEST-CHEMGRIDS, global gridded maps of the top 20 crop-specific pesticide application rates from 2015 to 2025. Sci Data. (2019) 6:170. doi: 10.1038/s41597-019-0169-4 31515508 PMC6761121

[48] Baker NT Stone WW. *Estimated annual agricultural pesticide use for counties of the conterminous United States 2008-12* (Nos. 2327-638X). Reston, Virginia, U.S.: US Geological Survey (2015).

[49] U.S. Geological Survey. Pesticide use maps - About the data. National Water-Quality Assessment (NAWQA) Project. . Available online at: https://water.usgs.gov/nawqa/pnsp/usage/maps/about.php (Accessed November 1, 2024).

[50] CropLife. CropLife top 100–2020 rankings. 2020 rankings of the largest ag retailers in the United States. (2021). Available online at: https://www.croplife.com/ (Accessed November 1, 2024).

[51] Taylor JR. An introduction to error analysis: the study of uncertainties in physical measurements, 2nd ed. New York, NY: University Science Books (1997).

[52] Theil H. A rank-invariant method of linear and polynomial regression analysis. Indagationes Mathematicae. (1950) 12:173.

[53] Wilcox RR. Fundamentals of modern statistical methods: Substantially improving power and accuracy, vol. 249. New York, NY, U.S.: Springer (2010). p. 207–210.

[54] Rao PV. In Statistical research methods in the life sciences. Pacific Grove, California, U.S.: Duxbury place (1998) p. 385–405.

[55] Benjamini Y Hochberg Y. Controlling the false discovery rate: a practical and powerful approach to multiple testing. J R Stat Society: Ser B (Methodological). (1995) 57:289–300. doi: 10.1111/j.2517-6161.1995.tb02031.x

[56] Skendžić S Zovko M Živković IP Lešić V Lemić D. The impact of climate change on agricultural insect pests. Insects. (2021) 12:440. doi: 10.3390/insects12050440 34066138 PMC8150874

[57] Coupe RH Capel PD. Trends in pesticide use on soybean, corn and cotton since the introduction of major genetically modified crops in the United States. Pest Manage Sci. (2016) 72:1013–22. doi: 10.1002/ps.4082 26194175

[58] Saleh R Bearth A Siegrist M. How chemophobia affects public acceptance of pesticide use and biotechnology in agriculture. Food Qual Preference. (2021) 91:104197. doi: 10.1016/j.foodqual.2021.104197

[59] DiBartolomeis M Kegley S Mineau P Radford R Klein K. An assessment of acute insecticide toxicity loading (AITL) of chemical pesticides used on agricultural land in the United States. PloS One. (2019) 14:e0220029. doi: 10.1371/journal.pone.0220029 31386666 PMC6684040

[60] Ambrus Á Yang YZ. Global harmonization of maximum residue limits for pesticides. J Agric Food Chem. (2016) 64:30–5. doi: 10.1021/jf505347z 25603277

[61] USDA-ERS. Ag and food statistics: charting the essentials. Agricultural Trade | Economic Research Service (2025). Available at: https://www.ers.usda.gov/data-products/ag-and-food-statistics-charting-the-essentials/agricultural-trade.

[62] MacLachlan DJ Hamilton D. Estimation methods for Maximum Residue Limits for pesticides. Regul Toxicol Pharmacol. (2010) 58:208–18. doi: 10.1016/j.yrtph.2010.05.012 20685381

[63] Rodriguez-Saona C Vincent C Isaacs R. Blueberry IPM: past successes and future challenges. Annu Rev Entomology. (2019) 64:95–114. doi: 10.1146/annurev-ento-011118-112147 30629894

[64] USDA-NASS. U.S. Department of Agriculture, National Agricultural Statistics Service. (N.d.). Parity index and prices paid and received by farmers (2025). Available online at: https://www.nass.usda.gov/Charts_and_Maps/Agricultural_Prices/parity.php (Accessed November 1, 2024).

[65] Wilson C Tisdell C. Why farmers continue to use pesticides despite environmental, health and sustainability costs. Ecol Economics. (2001) 39:449–62. doi: 10.1016/S0921-8009(01)00238-5

[66] Rosenheim JA Cass BN Kahl H Steinmann KP. Variation in pesticide use across crops in California agriculture: Economic and ecological drivers. *Science of The Total Environment* . 733. (2020) 138683. doi: 10.1016/j.scitotenv.2020.138683 32422460

[67] USDA-ERS. Adoption of genetically engineered crops in the United States—Recent trends in GE adoption. USDA Economic Research Service (2025). Available at: https://www.ers.usda.gov/data-products/adoption-of-genetically-engineered-crops-in-the-united-states/recent-trends-in-ge-adoption.

[68] Chahal PS Aulakh JS Jugulam M Jhala AJ. Herbicide-resistant Palmer amaranth (Amaranthus palmeri S. Wats.) in the United States—Mechanisms of resistance, impact, and management. Herbicides Agronomic Crops Weed Biol Rijeka Croatia: InTech. (2015), 1–29. doi: 10.5772/61512

[69] Price AJ Balkcom KS Culpepper SA Kelton JA Nichols RL Schomberg H. Glyphosate-resistant Palmer amaranth: A threat to conservation tillage. In J Soil Water Conserv (Vol. 66 Issue 4. (2011) pp:265–275). doi: 10.2489/jswc.66.4.265

[70] Duke SO. Why have no new herbicide modes of action appeared in recent years? Pest Manage Sci. (2012) 68:505–12. doi: 10.1002/ps.2333 22190296

[71] Gould F Brown ZS Kuzma J. Wicked evolution: Can we address the sociobiological dilemma of pesticide resistance? Science. (2018) 360:728–32. doi: 10.1126/science.aar3780 29773742

[72] Gatehouse AMR Ferry N Edwards MG Bell HA. Insect-resistant biotech crops and their impacts on beneficial arthropods. Philos Trans R Soc B: Biol Sci. (2011) 366:1438–52. doi: 10.1098/rstb.2010.0330 PMC3081576 21444317

[73] Lu Y Wu K Jiang Y Guo Y Desneux N. Widespread adoption of *Bt* cotton and insecticide decrease promotes biocontrol services. Nature. (2012) 487:362–5. doi: 10.1038/nature11153 22722864

[74] Gassmann AJ Reisig DD. Management of insect pests with *bt* crops in the United States. In Annu Rev Entomology. (2023) 68:31–49). doi: 10.1146/annurev-ento-120220-105502 36170641

[75] Gould F. Broadening the application of evolutionary based genetic pest management. In Evol (Vol. 62 Issue 2. (2008) pp:500–510). doi: 10.1111/j.1558-5646.2007.00298.x 17999722

[76] Hoy CW. Pesticide resistance management. In: Radcliffe EB Cancelado RE Hutchison &WD, editors. Integrated pest management: concepts, tactics, strategies and case studies. Cambridge, United Kingdom: Cambridge University Press (2008). p. 192–204.

[77] Harper CR Zilberman D. Pesticide regulation: Problems in trading off economic benefits against health risks. In: Zilberman D Siebert JB, editors. Economic perspectives on pesticide use in california. Berkeley, California: Division of Agriculture and Natural Resources, University of California (1990). p. 181–208.

[78] Palumbi SR. Humans as the world’s greatest evolutionary force. Science. (2001) 293:1786–90. doi: 10.1126/science.293.5536.1786 11546863

[79] Fernández-Ortuño D Grabke A Bryson PK Amiri A Peres NA Schnabel G. Fungicide resistance profiles in botrytis cinerea from strawberry fields of seven southern U.S. States. Plant Dis. (2014) 98:825–33. doi: 10.1094/PDIS-09-13-0970-RE 30708635

[80] Hawkins NJ Bass C Dixon A Neve P. The evolutionary origins of pesticide resistance. Biol Rev. (2019) 94:135–55. doi: 10.1111/brv.12440 PMC6378405 29971903

[81] Lucas JA Hawkins NJ Fraaije BA. The evolution of fungicide resistance. Adv Appl Microbiol. (2015) 90:29–92. doi: 10.1016/bs.aambs.2014.09.001 25596029

[82] Saito S Michailides TJ Xiao CL. Fungicide-resistant phenotypes in Botrytis cinerea populations and their impact on control of gray mold on stored table grapes in California. Eur J Plant Pathol. (2019) 154:203–13. doi: 10.1007/s10658-018-01649-z

[83] Bandara AY Weerasooriya DK Conley SP Bradley CA Allen TW Esker PD. Modeling the relationship between estimated fungicide use and disease-associated yield losses of soybean in the United States I: Foliar fungicides vs foliar diseases. PloS One. (2020) 15:e0234390. doi: 10.1371/journal.pone.0234390 32525917 PMC7289349

[84] Baltazar AR Santos FN Moreira AP Valente A Cunha JB. Smarter robotic sprayer system for precision agriculture. Electronics. (2021) 10:2061. doi: 10.3390/electronics10172061

[85] Dammer K-H Adamek R. Sensor-based insecticide spraying to control cereal aphids and preserve lady beetles. Agron J. (2012) 104:1694–701. doi: 10.2134/agronj2012.0021

[86] Terra FP Nascimento GH Duarte GA Drews-Jr PLJ. Autonomous agricultural sprayer using machine vision and nozzle control. J Intelligent Robotic Syst. (2021) 102:38. doi: 10.1007/s10846-021-01361-x

[87] Li Y Guo Z Shuang F Zhang M Li X. Key technologies of machine vision for weeding robots: A review and benchmark. Comput Electron Agric. (2022) 196:106880. doi: 10.1016/j.compag.2022.106880

[88] Martinez-Guanter J Agüera P Agüera J Pérez-Ruiz M. Spray and economics assessment of a UAV-based ultra-low-volume application in olive and citrus orchards. Precis Agric. (2020) 21:226–43. doi: 10.1007/s11119-019-09665-7

[89] Ayilara MS Adeleke BS Akinola SA Fayose CA Adeyemi UT Gbadegesin LA. Biopesticides as a promising alternative to synthetic pesticides: A case for microbial pesticides, phytopesticides, and nanobiopesticides. Front Microbiol. (2023) 14:1040901. doi: 10.3389/fmicb.2023.1040901 36876068 PMC9978502

[90] Patil V Jangra S Ghosh A. Advances in antisense oligo technology for sustainable crop protection. Crit Rev Plant Sci. (2024) 43:405–27. doi: 10.1080/07352689.2024.2394001

[91] Naranjo SE Ellsworth PC. Landscape considerations in pest management: Case study of the Arizona cotton IPM system. In Arthropod Manage Landscape considerations large-scale agroecosystems. (2024) Wallingford, UK: CABI, 44–77. doi: 10.1079/9781800622777.0003

[92] Raszick TJ. Boll weevil eradication: A success story of science in the service of policy and industry. In Ann Entomological Soc America (. (2021) 114:702–708). doi: 10.1093/aesa/saab031

[93] Figuera SG Babcock B Lubell M McRoberts N. Collective action in the area-wide management of an invasive plant disease. Ecol Soc. (2022) 27. doi: 10.5751/ES-13217-270212

[94] Haviland DR Stone-Smith B Gonzalez M. Control of pierce’s disease through areawide management of glassy-winged sharpshooter (Hemiptera: cicadellidae) and roguing of infected grapevines. J Integrated Pest Manage. (2021) 12:14. doi: 10.1093/jipm/pmab008

[95] Field to Market: The Alliance for Sustainable Agriculture. Trends in pest management in U.S. Agriculture: identifying barriers to progress and solutions through collective action. (2020) Washington, D.C.: Field to Market: The Alliance for Sustainable Agriculture, 68.

[96] Vekemans M-C Marchand PA. The european pesticides harmonised risk indicator HRI_1: A clarification about its displayed rendering. Eur J Risk Regul. (2024) 15:153–78. doi: 10.1017/err.2023.47

[97] Field to Market. Environmental outcomes from on-farm agricultural production in the United States (Fourth edition). Washington, D.C.: Field to Market: The Alliance for Sustainable Agriculture (2021), ISBN: 978-0-578-33372-4.

[98] Rosenheim JA Parsa S Forbes AA Krimmel WA Law YH Segoli M. Ecoinformatics for integrated pest management: expanding the applied insect ecologist’s tool-kit. J Economic Entomology. (2011) 104:331–42. doi: 10.1603/EC10380 21510177

[99] Eikenberry SE Iacona G Murphy EL Watson G Gerber LR. Identifying opportunities for high resolution pesticide usage data to improve the efficiency of endangered species pesticide risk assessment. Sci Total Environ. (2024) 921:170743. doi: 10.1016/j.scitotenv.2024.170743 38325484

[100] Kniss AR De Stefano A Arnold E Douglass C Myers C Paisley-Jones C. Honey bee toxicity of pesticides used in United States maize and soybean production, 1998–2020. In: Integrated environmental assessment and management (2025). doi: 10.1093/inteam/vjaf003 39987505

[101] U.S. EPA. How to comply with the 2015 revised worker protection standard for agricultural pesticides. In: U.S. Environmental protection agency office of pesticide programs (MC 7506C) 1200 pennsylvania ave. NW Washington: U.S. Environmental protection agency office of pesticide programs (2016). Available at: https://www.pesticideresources.org/migrated/wps/htc/htcmanual.pdf (Accessed November 1, 2024).

[102] U.S. EPA. Mitigation menu (2025). Available online at: https://www.epa.gov/pesticides/mitigation-menumitigation-options (Accessed November 1, 2024).

[103] Bamal D Duhan A Pal A Beniwal RK Kumawat P Dhanda S. Herbicide risks to non-target species and the environment: A review. Environ Chem Lett. (2024) 22:2977–3032. doi: 10.1007/s10311-024-01773-9

[104] Carlile B. Pesticides and non-target species. In: Pesticide selectivity, health and the environment. Cambridge University Press, Cambridge Core (2006). p. 211–244). doi: 10.1017/CBO9780511617874.013

[105] Heimpel GE Yang Y Hill JD Ragsdale DW. Environmental consequences of invasive species: greenhouse gas emissions of insecticide use and the role of biological control in reducing emissions. PloS One. (2013) 8:e72293. doi: 10.1371/journal.pone.0072293 23977273 PMC3748099

[106] U.S. EPA. U.S. Environmental protection agency protecting endangered species from pesticides (2024). Available online at: https://www.epa.gov/endangered-species (Accessed 12 December, 2024).

[107] Boudwin R Magarey R Jess L. Integrated pest management data for regulation, research, and education: crop profiles and pest management strategic plans. J Integrated Pest Manage. (2022) 13:13. doi: 10.1093/jipm/pmac011

