# Supplementary material for: Tracking sustainability in crop pest management in the United States using an eco-efficiency index
Source: Front Insect Sci. 2025 May 20;5:1582496. doi: 10.3389/finsc.2025.1582496 (PMC12130631; doi:10.3389/finsc.2025.1582496)
Supplement: Supplementary file 1 [file DataSheet1.zip › R Code.DOCX]

**S6. R code to calculate adjusted Theil-Sen estimates by species and crop group.**

library(readxl)

library(reshape2)

library(MASS) # boxcox and ginv

library(gnorm)

read_file_and_clean <- function(data_name){

clean_colnames <- c('year', 'fish', 'mammals', 'birds', 'aqua-inverts', 'pollinators',

'terr-plants', 'aqua-plants', 'terr-arthropods')

# Read in the data

data <- read_excel(paste0('raw/', data_name, '.xlsx'),

sheet = 'Base Log Summary',

range = 'J2:R29')

# Clean up the column names

colnames(data) <- clean_colnames

# Convert data from wide format to long format

data_long <- melt(data)

# Clean up the new column names; EEI = Eco-efficiency index

colnames(data_long) <- c('year', 'lifeform', 'log10_EEI')

# Make sure year is numeric

data_long$year <- as.numeric(data_long$year)

# Save the data as its own dataframe

return(data_long)

}

##########################################################################################

# Read in data and clean

# EEI = eco-efficiency index

##########################################################################################

corn_long <- read_file_and_clean('Corn')

soybean_long <- read_file_and_clean('Soybean') # Had to change name of sheet in excel file to read correctly

wheat_long <- read_file_and_clean('Wheat')

cotton_long <- read_file_and_clean('Cotton')

rice_long <- read_file_and_clean('Rice')

alfalfa_long <- read_file_and_clean('Alfalfa2')

veggies_fruits_long <- read_file_and_clean('VegFru')

orchards_grapes_long <- read_file_and_clean('OrcGra2') # original file not formatted in the same was as the others; made a new file and fixed that

pastures_hay_long <- read_file_and_clean('PasHay')

others_long <- read_file_and_clean('Other')

# Add in a column name for each crop

corn_long$crop <- 'corn'

soybean_long$crop <- 'soybean'

wheat_long$crop <- 'wheat'

cotton_long$crop <- 'cotton'

rice_long$crop <- 'rice'

alfalfa_long$crop <- 'alfalfa'

veggies_fruits_long$crop <- 'veggies_fruits'

orchards_grapes_long$crop <- 'orchards_grapes'

pastures_hay_long$crop <- 'pastures_hay'

others_long$crop <- 'other'

# Append all dataframes together

# looping through them isn't the best practice but given that

# the dataframes are pretty small it's ok

df_names <- c('corn_long', 'soybean_long', 'wheat_long', 'cotton_long',

'rice_long', 'alfalfa_long', 'veggies_fruits_long',

'orchards_grapes_long', 'pastures_hay_long', 'others_long')

df <- data.frame()

for(df_name in df_names){

eval(parse(text = paste0('df <- rbind(df, ', df_name, ')')))

}

# Change so that pollinators is the lifeform that is the baseline

df$lifeform <- factor(df$lifeform,

levels = c('pollinators', 'fish', 'mammals', 'birds',

'aqua-inverts', 'terr-plants', 'aqua-plants',

'terr-arthropods'))

# Add in a column that is the number of years from 1992,

# which is the baseline for the eco-efficiency index

df$years_from_1992 <- df$year - 1992

save(df, file = 'inter/all_crops_eei.RData')

##########################################################################################

# Check ANOVA assumptions

##########################################################################################

# Check if the normality assumption is met

hist(df$log10_EEI, probability = TRUE) # normality assumption not met

# Check if it's just one crop that's causing issues

for(df_name in df_names){

eval(parse(text = paste0('hist(', df_name, '$log10_EEI, probability = TRUE)')))

} # none of them appear to be normally distributed

# Transform the dependent variable in attempt to meet normality assumption

# boxcox requires that the response variable is positive first

df$EEI <- 10^(df$log10_EEI)

hist(df$EEI, probability = TRUE)

# Check if it's just one crop that's causing issues

for(crop in unique(df$crop)){

temp <- df[df$crop == crop,]

hist(temp$EEI, probability = TRUE, main = crop)

} # none of them appear to be normally distributed

box_cox_eei <- boxcox(df$EEI ~ df$crop + df$lifeform + df$year +

df$crop * df$lifeform + df$crop * df$year + df$lifeform * df$year +

df$crop * df$lifeform * df$year)

bc_lambda <- box_cox_eei$x[box_cox_eei$y == max(box_cox_eei$y)]

df$bc_eei <- (df$EEI^bc_lambda - 1)/bc_lambda

hist(df$bc_eei, probability = TRUE) # still not looking particularly normal

# Try taking the log (base e) of EEI and finding a different distribution

df$log_e_EEI <- log(df$EEI)

hist(df$log_e_EEI, probability = TRUE)

x <- seq(min(df$log_e_EEI), max(df$log_e_EEI), length.out = 100)

y <- dgnorm(x, alpha = 1.3, beta = 1.1)

hist(df$log_e_EEI, probability = TRUE)

lines(x,y)

##########################################################################################

# Calculate the Theil-Sen Estimator for each crop and lifeform

##########################################################################################

library(ggplot2)

library(latex2exp)

# Load in the file with all of the crops and lifeforms combined

load(file = 'inter/all_crops_eei.RData')

crops <- unique(df$crop)

lifeforms <- unique(df$lifeform)

for(crop in crops){

# Save the TS estimators for each crop separately

ts_estimators <- matrix(NA, nrow = length(lifeforms), 5)

ts_estimators_adj <- matrix(NA, nrow = length(lifeforms), 5)

colnames(ts_estimators) <- c('Slope', '95% CI LB', '95% CI UB', 't statistic', 'p-value')

rownames(ts_estimators) <- lifeforms

colnames(ts_estimators_adj) <- c('Slope', '95% CI LB', '95% CI UB', 't statistic', 'p-value')

rownames(ts_estimators_adj) <- lifeforms

# Calculate the TS estimators for each lifeform

j <- 1

for(lifeform in lifeforms){

# Subset the data to the correct crop and lifeform

crop_lifeform_df <- df[df$crop == crop & df$lifeform == lifeform,]

ymin <- min(crop_lifeform_df$log10_EEI, -1)

ymax <- max(crop_lifeform_df$log10_EEI, 3)

########################################

# Do not adjust for the fact that we're forcing the intercept to be zero

########################################

# what are all unique combinations of two pairs of points in the data

n <- nrow(crop_lifeform_df)

idxs <- combn(n, 2)

# Save the slopes for each combination of points

ts_slopes <- rep(NA, ncol(idxs))

for(i in 1:ncol(idxs)){

yj <- crop_lifeform_df$log10_EEI[idxs[2, i]]

yi <- crop_lifeform_df$log10_EEI[idxs[1, i]]

xj <- crop_lifeform_df$years_from_1992[idxs[2, i]]

xi <- crop_lifeform_df$years_from_1992[idxs[1, i]]

ts_slopes[i] <- (yj - yi)/(xj - xi)

}

# Calculate the final TS estimator, confidence interval, t statistic and p-value

crop_lifeform_TS <- median(ts_slopes)

ts_estimators[lifeform, 'Slope'] <- crop_lifeform_TS

# Calculate and plot the residuals

yhat <- crop_lifeform_df$years_from_1992*crop_lifeform_TS

resids <- crop_lifeform_df$log10_EEI - yhat

pdf(paste0('inter/resids/hists/', crop, '__', lifeform, '__resids.pdf'))

hist(resids)

dev.off()

# Calculate the standard error, based on the formula for the

# simple linear regression estimator

s2 <- sum(resids^2)/(n-1)

denom <- sum((crop_lifeform_df$years_from_1992 - mean(crop_lifeform_df$years_from_1992))^2)

se_ts <- sqrt(s2/denom)

# Calculate the confidence interval

t_star <- qt(0.975, df = n-1)

lb <- crop_lifeform_TS - t_star*se_ts

ub <- crop_lifeform_TS + t_star*se_ts

ts_estimators[lifeform, '95% CI LB'] <- lb

ts_estimators[lifeform, '95% CI UB'] <- ub

# Calculate the t statistic and p-value

tval <- crop_lifeform_TS/se_ts

pval <- 2*pt(abs(tval), df = n-1, lower.tail = FALSE)

ts_estimators[lifeform, 't statistic'] <- tval

ts_estimators[lifeform, 'p-value'] <- pval

# Plot the observed and the predicted values

ggplot(crop_lifeform_df) + geom_point(aes(x = years_from_1992, y = log10_EEI)) +

geom_abline(intercept = 0, slope = crop_lifeform_TS) +

xlab('Years since 1992') + ylab(TeX(r'($\log_{10}(EEI)$)')) +

ylim(c(ymin, ymax))

ggsave(paste0('inter/obs_vs_est/', crop, '__', lifeform, '__obs_vs_est.pdf'))

#####################################

# Adjust for the fact that we're forcing the intercept to be zero

# uses the standard error from normal linear regression as an

# estimate for the standard error for the slope

#####################################

# Just calculate the slope using (0,0) as the second point always

ts_slopes <- crop_lifeform_df$log10_EEI/crop_lifeform_df$years_from_1992

# Calculate the final TS estimator

crop_lifeform_TS <- median(ts_slopes, na.rm = TRUE)

ts_estimators_adj[lifeform, 'Slope'] <- crop_lifeform_TS

# Calculate and plot the histogram of the residuals

yhat <- crop_lifeform_df$years_from_1992*crop_lifeform_TS

resids <- crop_lifeform_df$log10_EEI - yhat

pdf(paste0('inter/resids/hists/', crop, '__', lifeform, '__resids__adjusted.pdf'))

hist(resids)

dev.off()

# Plot the residuals against time

crop_lifeform_df$resids <- resids

ggplot(crop_lifeform_df) + geom_point(aes(x = years_from_1992, y = resids)) +

xlab('Years since 1992') + ylab('Residual Value') +

ylim(c(ymin, ymax))

ggsave(paste0('inter/resids/scatter/adjresid_by_time__', crop, '__', lifeform, '.pdf'))

# Calculate the standard error, based on the formula for the

# simple linear regression estimator

s2 <- sum(resids^2)/(n-1)

denom <- sum((crop_lifeform_df$years_from_1992 - mean(crop_lifeform_df$years_from_1992))^2)

se_ts <- sqrt(s2/denom)

# Calculate the confidence interval

t_star <- qt(0.975, df = n-1)

lb <- crop_lifeform_TS - t_star*se_ts

ub <- crop_lifeform_TS + t_star*se_ts

ts_estimators_adj[lifeform, '95% CI LB'] <- lb

ts_estimators_adj[lifeform, '95% CI UB'] <- ub

# Calculate the t statistic and p-value

tval <- crop_lifeform_TS/se_ts

pval <- 2*pt(abs(tval), df = n-1, lower.tail = FALSE)

ts_estimators_adj[lifeform, 't statistic'] <- tval

ts_estimators_adj[lifeform, 'p-value'] <- pval

# Plot the observed and the predicted values

ggplot(crop_lifeform_df) + geom_point(aes(x = years_from_1992, y = log10_EEI)) +

geom_abline(intercept = 0, slope = crop_lifeform_TS) +

xlab('Years since 1992') + ylab(TeX(r'($\log_{10}(EEI)$)')) +

ylim(c(ymin, ymax))

ggsave(paste0('inter/obs_vs_est/', crop, '__', lifeform, '__obs_vs_est__adjusted.pdf'))

# update counter

j <- j + 1

}

# Save the final TS estimates, confidence intervals, t statistics, and p-values

write.csv(ts_estimators, file = paste0('output/final_ests_unadj/',

crop, '_ts_estimators.csv'))

write.csv(ts_estimators_adj, file = paste0('output/final_ests_adj/',

crop, '_ts_estimators.csv'))

}

#####################################

# Combine all Adjusted ts_estimator csvs to calculate a Benjamini-Hochberg p-value cutoff

#####################################

# Initialize dataframe that will contain all of the TS estimates,

# confidence intervals, t statistics, and p-values for every crop

all_adj_ts_stats <- read.csv('output/final_ests_adj/alfalfa_ts_estimators.csv',

col.names = c('Species', 'Slope', 'CI_LB_95',

'CI_UB_95', 't.statistic', 'p.value'))

all_adj_ts_stats$crop <- 'alfalfa'

crops_ex_alfalfa <- crops[crops != 'alfalfa']

# Add remaining crops

for(crop in crops_ex_alfalfa){

crop_ts_stats <- read.csv(paste0('output/final_ests_adj/',

crop, '_ts_estimators.csv'),

col.names = c('Species', 'Slope', 'CI_LB_95',

'CI_UB_95', 't.statistic', 'p.value'))

crop_ts_stats$crop <- crop

all_adj_ts_stats <- rbind(all_adj_ts_stats, crop_ts_stats)

}

# Sort by p-value

all_adj_ts_stats_sorted <- all_adj_ts_stats[order(all_adj_ts_stats$p.value),]

# Calculate the Benjamini-Hochberg cutoff using alpha = 0.05

all_adj_ts_stats_sorted$bh <- 0.05/(1:nrow(all_adj_ts_stats_sorted))

# Flag significant slopes after BH adjustment

all_adj_ts_stats_sorted$bh_sign_flag <- all_adj_ts_stats_sorted$p.value < all_adj_ts_stats_sorted$bh

# Determine the largest p-value that is still significant

bh_pval <- max(all_adj_ts_stats_sorted$p.value[all_adj_ts_stats_sorted$bh_sign_flag])

# Clean and sort to re-make table 2 (easier)

all_adj_ts_stats_sorted$Species <- factor(all_adj_ts_stats_sorted$Species,

levels = c('mammals', 'birds', 'terr-plants', 'terr-arthropods', 'pollinators',

'fish', 'aqua-inverts', 'aqua-plants'))

all_adj_ts_stats_sorted_clean <- all_adj_ts_stats_sorted[order(all_adj_ts_stats_sorted$crop,

all_adj_ts_stats_sorted$Species),

c('crop', 'Species', 'Slope', 'CI_LB_95',

'CI_UB_95', 't.statistic', 'p.value', 'bh_sign_flag')]

write.csv(all_adj_ts_stats_sorted_clean, file = 'output/final_ests_adj/bh_ts_estimators.csv',

row.names = FALSE)
