# Supplementary material for: Tracking sustainability in crop pest management in the United States using an eco-efficiency index
Source: Front Insect Sci. 2025 May 20;5:1582496. doi: 10.3389/finsc.2025.1582496 (PMC12130631; doi:10.3389/finsc.2025.1582496)
Supplement: Supplementary file 1 [file DataSheet1.zip › Supplementary Methods.DOCX]

**S1. Supplementary Information on Methods**

Methods for Collecting NASS Production Data

**Methods for Collecting NASS Production Data Using QuickStats**

To estimate the annual production of crops for corn, wheat, rice, “aggregated pasture and hay”, and alfalfa, we collected publicly available data from the USDA National Agricultural Statistics (NASS) website using the “Quick Stats” database. This database allows for targeted searches on specific commodities, categories, measurement units, and locations.

**Methods for Collecting NASS Production Data Using Crop Summary Reports**

To gather data for the “orchard and grape” category, we collected data from both the annual “USDA NASS Citrus Fruits Summary” report and the annual “USDA NASS Non-Citrus Fruits and Nuts Summary”. The “USDA NASS Citrus Fruits Summary” contained the following crops: K-early citrus fruit, lemons, limes, oranges, tangelos, tangerines, temples, and mandarins while the “USDA NASS Non-Citrus Fruits and Nuts Summary” contained data on apples, apricots, sweet cherries, tart cherries, dates (California), figs (California), grapes, kiwifruit (California), nectarines (California), olives (California), papayas (Hawaii), peaches, pears, plums (California), prunes (California), prunes and plums, as well as nuts like almonds, hazelnuts, macadamias, pecans, pistachios, and walnuts. Additionally, it includes citrus fruits like oranges and grapefruits.

The “vegetable and fruit” category was primarily sourced from the annual “USDA Non-Citrus Fruits and Nuts Summary” report and the annual “USDA NASS Vegetable Summary”. Additional crops within the “vegetable and fruit” category, including dry beans and peas, potatoes, and sweet potatoes, required data collection using the “Quick Stats” database. The vegetable summary included the following crops with some variation year to year: artichokes, asparagus, snap beans, broccoli, cabbage, cantaloupes, carrots, cauliflower, celery, sweet corn, cucumbers, garlic, honeydews, lettuce (head, leaf, and romaine), onions, bell peppers, chile peppers, pumpkins, spinach, squash, tomatoes, and watermelons.

**Variation in Production Measurements**

Each crop has unique measurement units: corn, wheat, and soybeans are measured in bushels; cotton in 480-pound bales; rice in hundredweight (CWT); and “aggregated pasture and hay” and alfalfa are measured in tons. The crop categories containing multiple crops (“orchard and grapes”, “vegetable and fruits”, and “aggregated other crops”) were provided in a variety of measurement units. All crop categories were converted to metric units for analytical purposes.

**Changes in Vegetable Production Reporting Method**

One issue we encountered was the changes in data reporting over time. From 1992 to 2015, vegetable totals were recorded as "Principal Vegetable", whereas, from 2016 to 2018 they were reported as "Principle Vegetable Fresh Market and Processing", nearly doubling the overall production total. Additionally, between 1992 and 1995, all berries (under the vegetable and fruit category) were recorded as a single unit. However, after 1995, production was reported separately as blueberries, cranberries, and caneberries thereafter. The most significant changes in data reporting and categorization, including missing data for certain years, affected the “vegetable and fruit” and “orchard and grapes” categories.

**Vegetable and Fruit Category:**

- No available data on the following crops in NASS that are included in the USGS crop list for Vegetables and Fruits: beets, chicory, currants, and eggplant.
- Incomplete data not included in analysis: eggplant, ginger root, herbs/spices, okra, bananas/plantains, pineapples, root tubers (cassava, yautia, yam), turnips(forage)
- Strawberry production totaled listed in annual “USDA Vegetable Summary” - listed separately from vegetable totals.
- Celery is included in “USDA Vegetable Totals” but not USGS crop list

**Orchard and Grapes Category:**

- No available data on the following crops in NASS that are included in the USGS crop list for “Orchard and Grapes”: chestnuts
- Incomplete data not included in analysis: mango, persimmons, certain nuts (brazil, nutmeg, cashew, groundnuts).
- Stone-like fruit data includes nectarines.
- Production only reported for California: dates, figs, nectarines, olives, plums, prunes
- Production only reported for Hawaii: papaya

Methods for the addition of CAS numbers and pesticide use type to pesticide data

**CAS numbers for the Total Applied toxicity data** were obtained from the U.S. EPA from the following source: <https://gaftp.epa.gov/COMPTOX/Sustainable_Chemistry_Data/Chemistry_Dashboard/CPDat/CPDat2020-12-16/> and added to the Total Applied Toxicity and pesticide use data.

The data as a CSV file was split into four ~1000000 line files that MS Excel could load in their entirety and wrote the following function:

=IFNA(VLOOKUP(B2&"*",split_1!$B$2:$E$1000000,4,FALSE),IFNA(VLOOKUP(B2&"*",split_2!$B$2:$E$1000000,4,FALSE),IFNA(VLOOKUP(B2&"*",split_3!$B$2:$E$1000000,4,FALSE),IFNA(VLOOKUP(B2&"*",split_4!$B$2:$E$911921,4,FALSE),""))))

Using nested IFNA functions, this takes the chemical name (specifically names beginning with the chemical name, hence the B2&"*") from the spreadsheet and checks it against the name in the CSV files (Split_*), then fills the respective CAS number cell in the spreadsheet with the preferred CAS number from the CSV. Any blank cells were manually checked.

**Pesticide use type data** was obtained from the [National IPM Database.](https://ipmdata.ipmcenters.org/)  A lookup function was used to create a match for the chemical names in the pesticide use data files. Where multiple types were found for a single pesticide ai, the most widely used type was listed. If a pesticide AI was listed as fumigant plus other uses it was considered to be a fumigant.
