# Supplementary figures and images for: Tracking sustainability in crop pest management in the United States using an eco-efficiency index
Source: Front Insect Sci. 2025 May 20;5:1582496. doi: 10.3389/finsc.2025.1582496 (PMC12130631; doi:10.3389/finsc.2025.1582496)

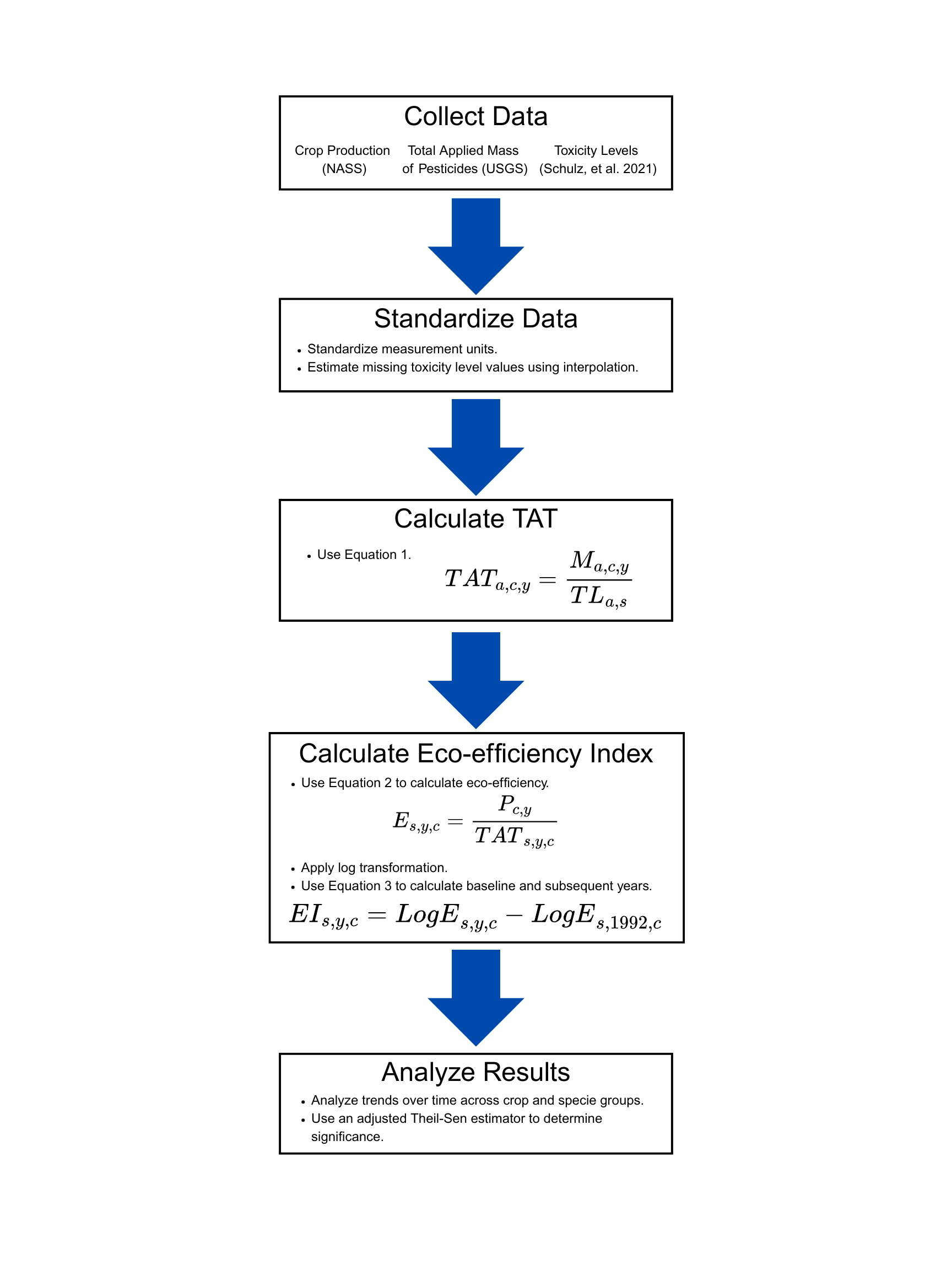

Supplement: Supplementary file 1 [file DataSheet1.zip › Supplementary Figure S1.PNG]
